# Gaining new insights into the etiology of ulcerative colitis through a cross-tissue transcriptome-wide association study

**DOI:** 10.3389/fgene.2024.1425370

**Published:** 2024-07-18

**Authors:** Shijie Ren, Chaodi Sun, Wenjing Zhai, Wenli Wei, Jianping Liu

**Affiliations:** ^1^ Graduate School, Hebei University of Chinese Medicine, Shijiazhuang, Hebei, China; ^2^ Department of Gastroenterology, The First Affiliated Hospital of Hebei University of Chinese Medicine, Shijiazhuang, Hebei, China

**Keywords:** genetic structure, pathogenic mechanism, polymorphism, TWAS, ulcerative colitis

## Abstract

**Background:**

Genome-wide association studies (GWASs) have identified 38 loci associated with ulcerative colitis (UC) susceptibility, but the risk genes and their biological mechanisms remained to be comprehensively elucidated.

**Methods:**

Multi-marker analysis of genomic annotation (MAGMA) software was used to annotate genes on GWAS summary statistics of UC from FinnGen database. Genetic analysis was performed to identify risk genes. Cross-tissue transcriptome-wide association study (TWAS) using the unified test for molecular signatures (UTMOST) was performed to compare GWAS summary statistics with gene expression matrix (from Genotype-Tissue Expression Project) for data integration. Subsequently, we used FUSION software to select key genes from the individual tissues. Additionally, conditional and joint analysis was conducted to improve our understanding on UC. Fine-mapping of causal gene sets (FOCUS) software was employed to accurately locate risk genes. The results of the four genetic analyses (MAGMA, UTMOST, FUSION and FOCUS) were combined to obtain a set of UC risk genes. Finally, Mendelian randomization (MR) analysis and Bayesian colocalization analysis were conducted to determine the causal relationship between the risk genes and UC. To test the robustness of our findings, the same approaches were taken to verify the GWAS data of UC on IEU.

**Results:**

Multiple correction tests screened PIM3 as a risk gene for UC. The results of Bayesian colocalization analysis showed that the posterior probability of hypothesis 4 was 0.997 and 0.954 in the validation dataset. MR was conducted using the inverse variance weighting method and two single nucleotide polymorphisms (SNPs, rs28645887 and rs62231924) were included in the analysis (*p* < 0.001, 95%CI: 1.45-1.89). In the validation dataset, MR result was *p* < 0.001, 95%CI: 1.19-1.72, indicating a clear causal relationship between PIM3 and UC.

**Conclusion:**

Our study validated PIM3 as a key risk gene for UC and its expression level may be related to the risk of UC, providing a novel reference for further improving the current understanding on the genetic structure of UC.

## 1 Introduction

Ulcerative colitis (UC) is a chronic inflammatory disease of the colon that can affect individuals of any age but is most common to those aged between 20 and 40 years old (Voelker, 2024). UC has a high morbidity rate, imposing huge burden to the healthcare system. The epidemiology of UC has changed over the past few decades, with developed Western countries showing a relatively high prevalence rate (Ng et al., 2017). In developing regions such as South America, Asia, Africa, and Eastern Europe, the incidence of UC is also steadily increasing (Aniwan et al., 2017; Shouval and Rufo, 2017). UC is related to immune and genetic factors and it will increase the risk of developing intestinal tumors. At present, the mechanism of the disease is not clear and we still face a lack of effective treatment. Therefore, a better understanding of the underlying mechanisms of UC is required to help develop effective therapeutic targets.

Family and twin studies revealed a genetic susceptibility to the pathogenesis of UC. The prevalence of UC among the relatives of UC patients is significantly higher than that in the background population (Annese, 2020). Genome-wide association studies (GWASs) have identified at least 133 UC-associated loci (Wang et al., 2014), but the exact roles of many loci still remained unknown. Most GWAS signals are located in non-coding regions, which often overlap with gene regulatory elements and highly enriched expression quantitative trait loci (eQTL), suggesting that transcriptional regulation plays a crucial role in influencing UC susceptibility.

Multi-marker analysis of genomic annotation (MAGMA) is a fast and flexible tool for gene and gene set analysis based on GWAS data (de Leeuw et al., 2015). MAGMA uses multiple regression methods to effectively integrate linkage disequilibrium (LD) between variants to discover multi-variant effects. Moreover, this approach also enables pathway analysis and detection of genes and pathways associated with disease risk based on gene set analysis using gene-level regression models (Sniekers et al., 2017). Transcriptome-wide association study (TWAS) combining eQTL and GWAS data could be used to identify genes that influence the complex traits and diseases through genetic regulation of gene expressions (Wainberg et al., 2019; Strunz et al., 2020). TWAS has been successfully applied to screen risk genes for a variety of complex human diseases, such as Alzheimer’s disease (Hao et al., 2018; Luningham et al., 2020), cardiovascular disease (Theriault et al., 2019), etc., Most of the current TWAS studies calculate the genetic expression matrix in each tissue, but this may overlook shared local regulation of gene expression. Some eQTLs have been confirmed to be able to regulate gene expression in different tissues (Liu et al., 2017). To overcome such a problem, the unified test for molecular signatures (UTMOST) has been developed to conduct cross-tissue gene-level association analysis. Higher statistical efficiency (Hu et al., 2019) of cross-tissue association analysis allows it to be increasingly applied to study complex diseases, such as inflammatory bowel disease, schizophrenia, etc., (Uellendahl-Werth et al., 2022). Fine-mapping of causal gene sets (FOCUS) is a fine-mapping method that estimates a set of potentially causative genes by prioritizing null models in null simulations using predicted eQTL weights, LD, and GWAS summary statistics, and accurately identifies disease-causing genes when genes in a certain region affect downstream traits (Mancuso et al., 2019). It has been well used in the studies of Parkinson’s disease and lipid metabolism (Mancuso et al., 2019; Shi et al., 2024).

In this study, MAGMA was used for gene annotation, and cross-tissue analysis of UC was conducted based on integrated data of the eQTL data (GTExv8) of the Genotype Tissue Expression (GTEx) project (https://www.gtexportal.org/) and the GWAS data of UC in the FinnGen database (https://storage.googleapis.com/finngen-public-data-r9/summary_stats/finngen_R9_K11_UC_STRICT2.gz). In addition, functional summary-based imputation (FUSION), conditional and joint analysis and FOCUS were used to process the GWAS data of UC to screen risk genes. Reliable UC risk genes were obtained by taking the intersection of the results of the four analyses. In order to verify the robustness of the results, a GWAS data set of UC on IEU (https://gwas.mrcieu.ac.uk/) was utilized to conduct the same analyses.

## 2 Methods

### 2.1 GWAS data source of UC

GWAS data of UC, which included 5,034 cases and 371,530 controls of European population, were obtained from the FinnGen database. The subjects in this dataset were strictly diagnozed as having UC and had data provided by Finland’s Kansaneläkelaitos (KELA). There were at least two health data repositories (HDRs) in this dataset at the same time. Informed consent, quality control and other information can be found in published papers.

### 2.2 MAGMA for gene annotation

MAGMA software is a gene and pathway analysis tool based on multivariable regression models (Yuan et al., 2024). MAGMA uses multiple regression models to calculate the cumulative effect of multiple single nucleotide polymorphisms (SNPs assigned to a specific gene (±10 kb) but with better statistical power than other tools (de Leeuw et al., 2015). LD was calculated using data from the 1,000 Genomes European population as a reference panel (Genomes et al., 2015). To detect biologically relevant pathways in UC etiology, MAGMA-based gene set analysis was used. In MAGMA, gene set analysis was built in a linear regression model using the *p*-values of genes and gene correlation matrices. BioCarta, KEGG and Reactome pathways were downloaded from MSigDB data (https://www.gsea-msigdb.org/gsea/msigdb).

### 2.3 Cross-tissue and single-tissue TWAS analysis

Based on the results of gene annotation, risk genes were further identified. Here, three genetic analysis methods (UTMOST, FUSION and conditional and joint analysis) were used rationally. The GWAS data and eQTL data of UC from 44 tissues in GTExV8 were integrated to estimate the genetic component of gene expression in each tissue. To analyze the association between genes and diseases, the UTMOST was employed for TWAS analysis to obtain TWAS results for individual tissues in GTExV8. Next, based on the single-tissue analysis, cross-tissue TWAS analysis was performed using we used UTMOST to calculate the cross-tissue joint test correlation results in GTExV8. For each gene, UTMOST trains a cross-tissue expression imputation model based on a penalised multivariate regression that accounts for different directions and effect sizes of eQTL signals across tissues (Ni et al., 2022). To reduce the risk of noise and false positive rates in UTMOST cross-tissue association testing, we incorporated the UC GWAS and eQTL data of whole blood from GTExv8 using the FUSION software for validation (Gusev et al., 2016). FUSION uses a variety of penalised linear models (GBLUP, LASSO, etc.) to build prediction models for significant cis-genetic genes estimated by SNPs within 500 kb on both sides of the gene boundary and then selects the best model based on the prediction results. False discovery rate (FDR) < 0.05 (after Benjamini–Hochberg correction) indicated a significant TWAS result for both cross-tissue and single-tissue analyses.

### 2.4 Conditional and joint analysis

The problem of only selecting the most significant SNP in a region could be solved by performing conditional and joint analysis, which uses summary-level statistics from a meta-analysis of GWAS and estimates LD from a reference sample with individual-level genotype data (Yang et al., 2012). Genome-wide FDR-corrected conditional joint analysis of significant TWAS signals was used to evaluate GWAS association signals after removing TWAS association signals. We selected SNPs based on *P*_FDR_ < 0.05 and estimated the joint effect of all the selected SNPs after model optimization.

### 2.5 FOCUS for precise gene location

FOCUS software was used for fine-mapping transcriptome-wide correlation study statistics to genomic risk regions. The software aggregates GWAS data and eQTL weights as inputs and a set of credible genes as outputs to interpret the observed genomic risk (Mancuso et al., 2019). FOCUS provides a pre-built database of weights from multiple tissues, multiple eQTL reference combinations, including GTExv8 weights from PrediXcan. In our study, risk genes were screened based on the Marginal posterior inclusion probability (PIP) of 0.8 and *P* < 5e-8.

### 2.6 The intersection of the four analysis results and MR analysis

The risk genes obtained by the above four analyses (MAGMA, UTMOST, FUSION and FOCUS) were intersected to obtain key genes, which were then subjected to Mendelian randomization (MR) analysis and Bayesian colocalization analysis.

MR is a causal inference method that indirectly evaluates the causal relationship between exposure and clinical outcomes by using genes closely related to certain specific traits as instrumental variables (IVs) to replace exposure factors in the regression model (Hemani et al., 2018). To identify eligible IVs, three key assumptions must be met. Specifically, Assumption one states that genetic variation is directly associated with exposure. To achieve this, we defined SNPs as being directly associated with the exposure at *P* < 5E-08 (genome-wide significance threshold). Assumption two states that genetic variation should not be directly related to confounding factors. Assumption 3 states that genetic variation should not be directly related to outcomes. The latter two hypotheses are manifested as horizontal pleiotropy in post-MR (Hemani et al., 2018).

Qualified IVs were clustered within a distance of 10,000 kb with an LD of r^2^ < 0.3. Following this, IVs were extracted from the outcome features and these IVs were harmonized in exposure and outcome GWAS datasets. Finally, Wald ratio was used in the MR analysis method if only one independent IV was included, and if two or more IVs were included, inverse variance weighting (IVW) was used in the MR analysis method (Sanderson et al., 2022). *p* < 0.05 was considered as statistically significant. When 3 or more IVs were available, sensitivity analyzes including MR-Egger, weighted mode, weighted median mode, and simple mode analysis were performed to assess the robustness of the results. The horizontal pleiotropy (MR Egger’s intercept test), heterogeneity (Cochran’sQ test) and outliers (MR-PRESSO test) (Hemani et al., 2018) were tested by post-MR analysis. Finally, as only 2 IVs were obtained and we therefore could not perform subsequent sensitivity analysis or post-MR analysis. The above-mentioned analysis approaches were all implemented in the R package “TwoSampleMR”.

### 2.7 Bayesian colocalization analysis

Bayesian colocalization analysis could be use to estimate whether two associated signals are consistent with shared causal variation (Giambartolomei et al., 2014). The COLOC package in R (with default parameters) was used for analysis and for testing the posterior probabilities of 5 hypotheses as follows: H0: phenotype 1 and phenotype 2 are not significantly associated with any SNP locus in a genomic region; H1: associated with phenotype 1 but not with phenotype 2; H2: associated with phenotype 2 but not with phenotype 1; H3: phenotype 1 and phenotype 2 are significantly associated with a SNP loci in a genomic region but are driven by different causal variant loci; H4: phenotype 1 and phenotype 2 are significantly associated with a SNP loci in a genomic region driven by the same causal loci. The posterior probability of hypothesis 4 (PPH4) > 0.8 is considered to be able to indicate that the two associated signals are consistent with a shared causal variant (Giambartolomei et al., 2014).

### 2.8 Validation of genetic analysis results using GWAS data from IEU

To explore the robustness of the risk genes for UC obtained by the above analyses, the UC GWAS data (ieu-a-32) on the IEU OpenGWAS project (https://gwas.mrcieu.ac.uk/) was processed for verification following the same steps as above. The GWAS dataset contained 6,968 cases and 20,464 controls of European population.

## 3 Results

### 3.1 Gene-based association study and pathway enrichment analysis

**FIGURE 1:**
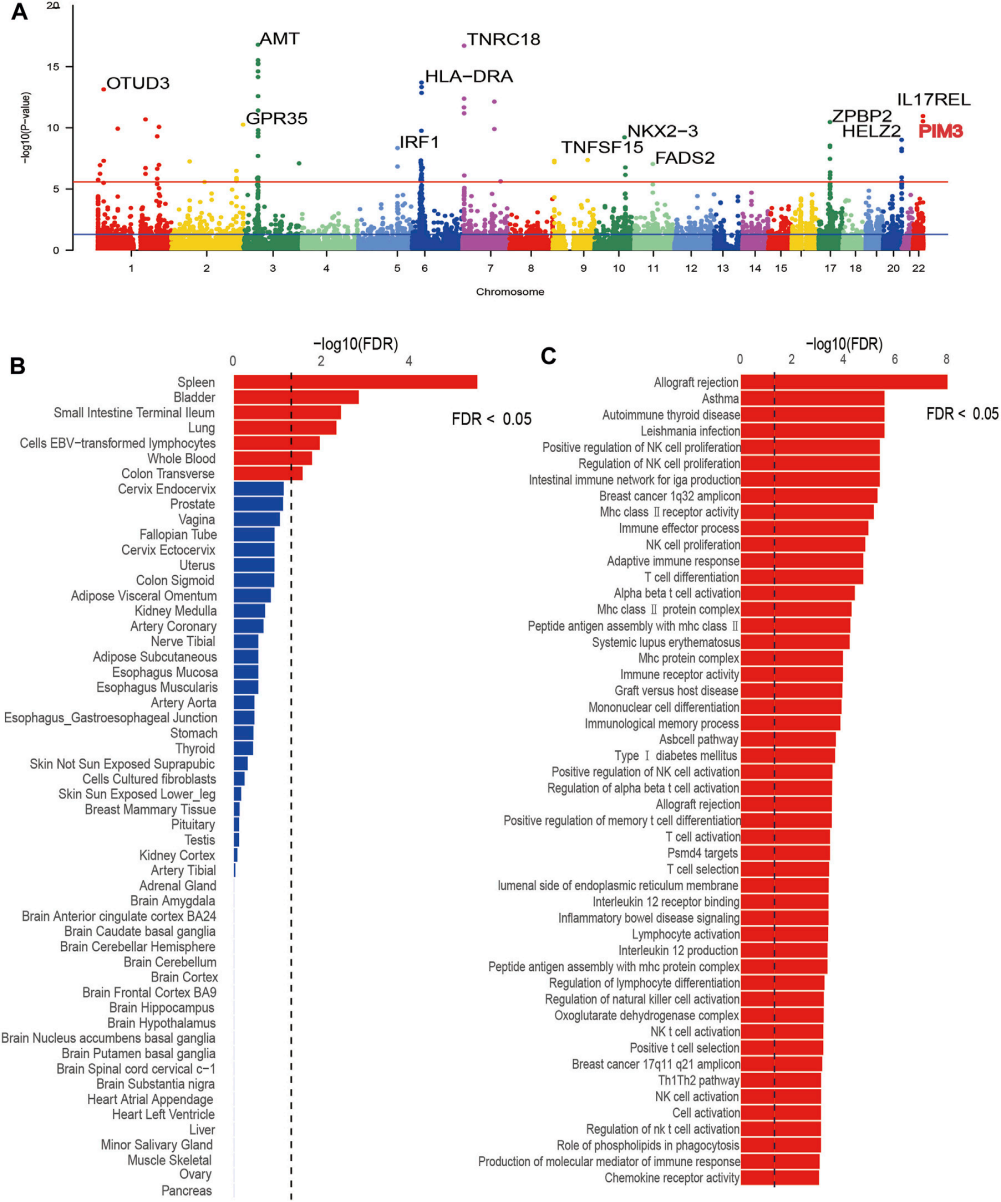
Results plot after MAGMA software screening. **(A)** is a Manhattan plot marking significant genes for each chromosome, **(B)** is the tissue-specific enrichment results, and **(C)** is the pathway enrichment results.

MAGMA was used to annotate UC-related risk genes. A total of 412 significant genes were obtained after FDR correction (*p* < 0.05) (Supplementary Table S1), and then the most important genes in each chromosome were labeled in a Manhattan plot (Figure 1A). In terms of tissue-specific enrichment (Figure 1B), a total of seven tissues showed positive results after FDR correction (*p* < 0.05) and the terminal ileum of the small intestine, transverse colon, and whole blood can be seen. Pathway enrichment analysis by MAGMA identified a total of 165 significantly enriched gene sets (*P*_FDR_ < 0.05), and Figure 1C displayed the top 50 pathways with the most significant *p*-values. Among the top 50 significant pathways, there were pathway signals such as transplant rejection, asthma, autoimmune thyroid disease, regulation of natural killer cell (NK cell) proliferation, and IgA production in the intestinal immune network. These pathways were closely related to immune diseases and inflammatory diseases, suggesting that the risk genes annotated by MAGMA were reliable. In the validation dataset, 350 significant genes were obtained after FDR correction (*p* < 0.05) (Supplementary Table S2). Pathway enrichment analysis revealed a total of 70 significant pathways after FDR correction (*p* < 0.05), and the top-ranking pathways included those related to inflammatory bowel disease, immune cells, inflammatory factors, and others (Supplementary Figure S1). Tissue-specific enrichment analysis detected a total of significant eight tissues after FDR correction (*p* < 0.05) (Supplementary Figure S2), including whole blood and intestinal tissues. The trends in the validation dataset were consistent with the current results.

### 3.2 Transcriptome-wide association study results of UC

**FIGURE 2:**
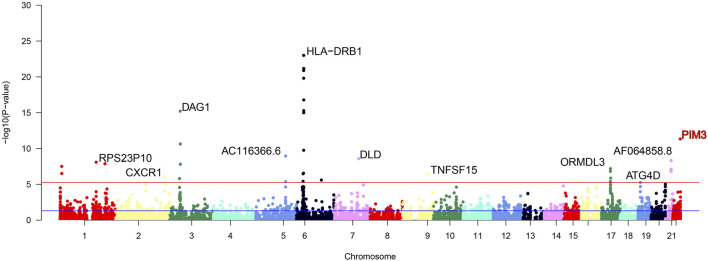
Manhattan plot of significant genes after FUSION screening. The Manhattan plot displays significant genes identified after FUSION screening, with the topmost gene labeled as the most significant. Gene highlighted in red is the focus of this study.

Cross-tissue findings revealed that a total of 35 genes showed statistically significant signals after FDR correction (*p* < 0.05) (Table 1). For single-tissue internal validation, 148 of all 8,756 genes modeled in the genotype data with significant cis-genetic expression in whole blood of the GTExv8 dataset had significant TWAS association signals after FDR correction (*p* < 0.05) (Supplementary Table S3). The Manhattan plot displayed the most significant genes on each chromosome (Figure 2). In summary, six overlapping candidate genes were identified by cross-tissue and single-tissue testing (Supplementary Table S4). In the validation dataset, after FDR correction (*p* < 0.05), the cross-tissue analysis screened a total of 28 significant genes (Supplementary Table S5). In the single-tissue analysis, a total of 178 significant genes were identified after FDR correction (*p* < 0.05, Supplementary Table S6). There were 5 common genes exsiting in all the results of the above analyses (Supplementary Table S7).

**TABLE 1:** The significant genes for UC risk in cross-tissue TWAS analysis.

Gene	Chr	Test score	*P*	*P*_FDR_
SATB2	2	21.22	7.82E-10	1.90E-06
AC021016.7	2	20.28	1.02E-09	1.90E-06
PIM3	22	17.22	8.36E-09	1.04E-05
ARPC2	2	15.43	5.94E-08	5.55E-05
PNKD	2	15.72	1.03E-07	7.72E-05
WNT10A	2	15.48	2.53E-07	1.57E-04
CXCR2	2	12.79	1.05E-06	5.61E-04
TNS1	2	13.10	1.89E-06	8.81E-04
PASK	2	11.80	2.53E-06	8.89E-04
TMEM163	2	12.16	2.36E-06	8.89E-04
COPS9	2	12.89	2.62E-06	8.89E-04
LINC01494	2	12.59	2.95E-06	9.18E-04
KIAA1841	2	11.39	5.16E-06	1.48E-03
CNOT11	2	11.29	1.13E-05	3.02E-03
HSPD1	2	11.27	1.58E-05	3.93E-03
BIN1	2	10.48	1.76E-05	4.04E-03
CTDSP1	2	10.43	1.84E-05	4.04E-03
GPR35	2	9.06	2.08E-05	4.31E-03
AC097468.4	2	10.66	2.71E-05	5.05E-03
SATB2-AS1	2	10.33	2.70E-05	5.05E-03
CCNT2-AS1	2	9.96	3.42E-05	6.07E-03
CCNT2	2	8.19	5.04E-05	8.55E-03
RP11-681L4.1	2	52.97	5.90E-05	9.58E-03
FAP	2	9.47	6.67E-05	1.03E-02
IFIH1	2	9.19	7.49E-05	1.11E-02
MPP4	2	8.86	1.33E-04	1.91E-02
ETNPPL	4	8.79	1.42E-04	1.96E-02
DUSP28	2	8.57	1.61E-04	2.15E-02
AAMP	2	7.99	1.88E-04	2.42E-02
RNA5SP122	2	19.52	2.70E-04	3.36E-02
RP11-399F2.2	4	−3.63	2.81E-04	3.39E-02
AC016747.3	2	7.05	2.99E-04	3.49E-02
PUS10	2	6.92	3.27E-04	3.71E-02
AC017002.2	2	7.58	3.77E-04	4.15E-02
HORMAD2	22	7.32	3.94E-04	4.21E-02

UC, ulcerative colitis; TWAS, transcriptome-wide association study.

NOTE: “Test score” refers to the evaluation score obtained from the UTMOST software.

### 3.3 Conditional and joint analysis

**FIGURE 3:**
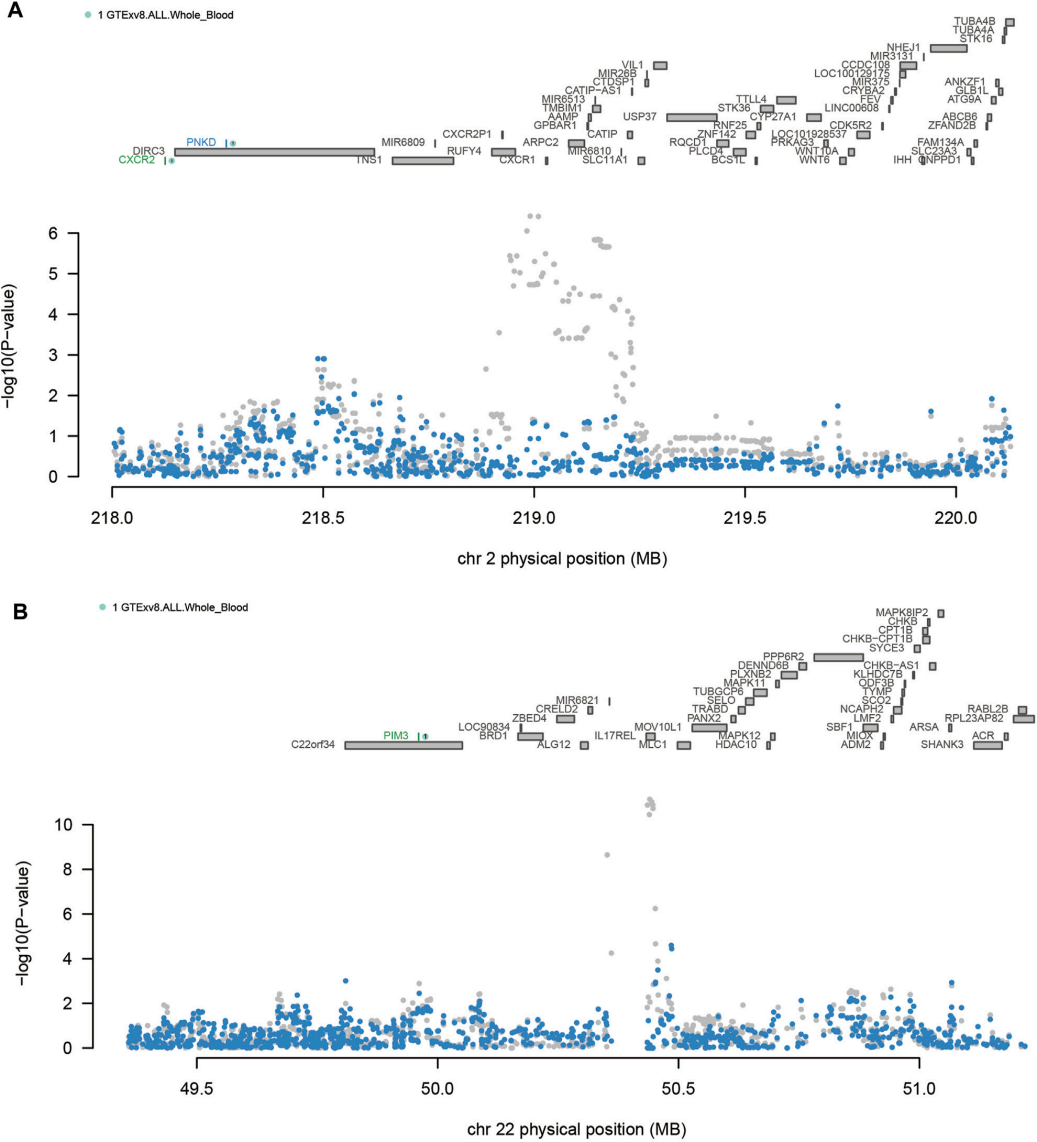
Regional associations of transcriptome-wide association study (TWAS) hits. **(A)** Association plot of the chromosome 2q35 region. **(B)** Association plot of the chromosome 22q13.33 region. The top panel highlights all genes in this region. Marginally related TWAS genes are shown in blue, and co-significant genes are shown in green. The figure shows a Manhattan area plot of genome-wide association study (GWAS) data before (gray) and after (blue) regulating the predicted expression of a green gene.

As shown in Table 2 4 loci, namely, 2q21.3 (TMEM163), 2q35 (CXCR2), 2q37 (DUSP28) and 22q13.33 (PIM3) (conditional *p* < 0.05), represented independent signals of multiple important genes. We observed that some GWAS signals were driven by genetically regulated gene expression. For example, CXCR2 accounted for most of the signal at 2q35 locus, whereas the TWAS signal of PNKD was significantly reduced if conditioned on the predicted expression of CXCR2 (Figure 3A). Similarly, PIM3 accounted for most of the signal at the 22q13.33 locus (Figure 3B). In the validation dataset, there were two loci [2q35 (CXCR1) and 22q13.33 (PIM3)] representing independent signals for multiple important genes (Supplementary Table S8).

**TABLE 2:** The significant genes for UC risk in Conditional and joint analysis.

Gene	TWAS.Z	TWAS.*P*	Joint.Z	Joint.*P*
CXCR2	−4.90	8.90E-07	−4.90	8.90E-07
TMEM163	4.40	1.00E-05	4.40	1.00E-05
DUSP28	−3.70	2.10E-04	−3.70	2.10E-04
PIM3	6.90	4.60E-12	6.90	4.70E-12

NOTE: Joint.Z is the Z value after conditional joint analysis, and Joint.*P* is the *P* value after 621 conditional joint analysis.

### 3.4 The results of FOCUS precision positioning

**FIGURE 4:**
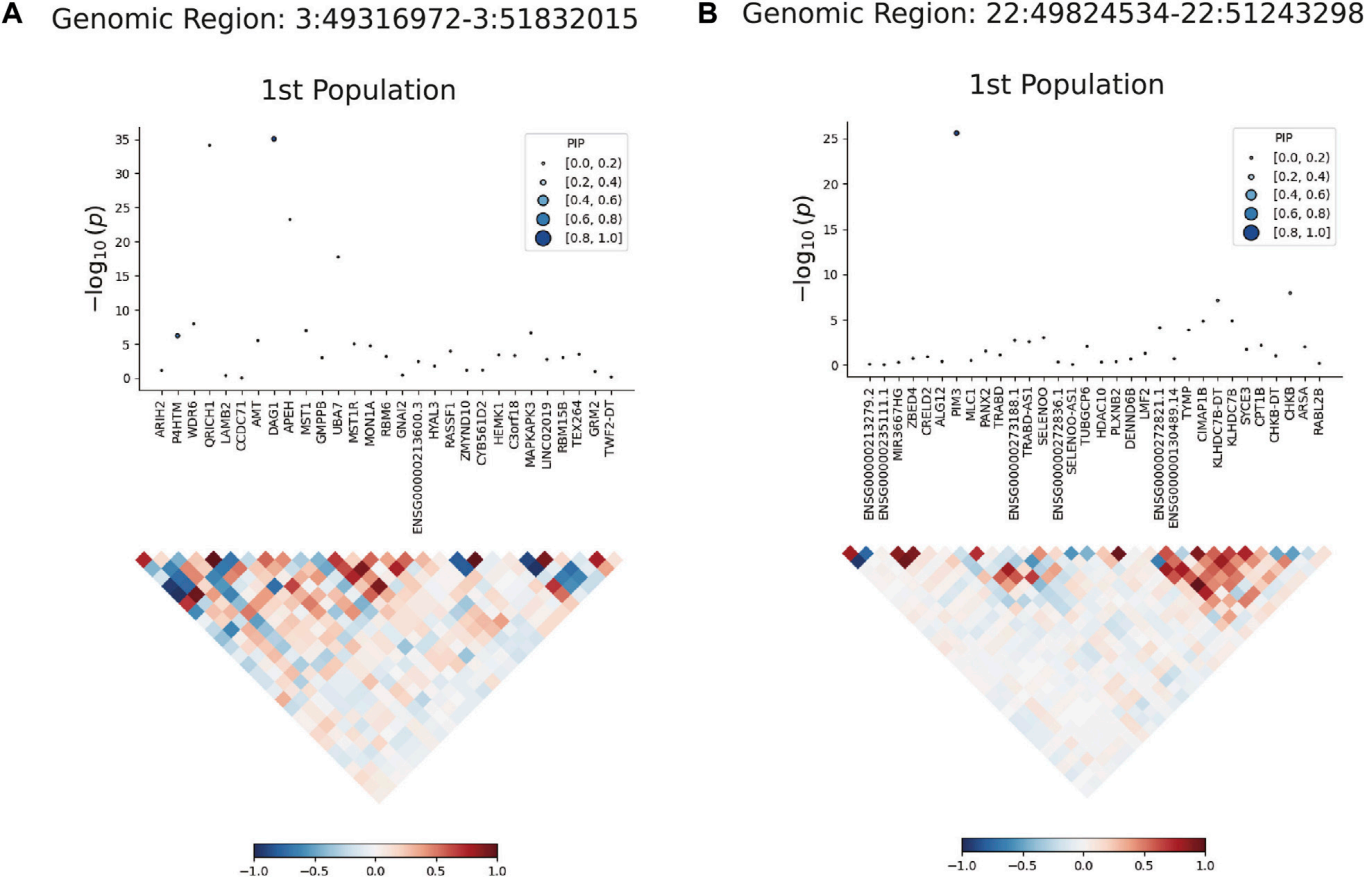
Results plot after FOCUS software precision positioning. PIP, Posterior Inclusion Probability. **(A)** shows the most significant gene DARG1 in the region of chromosome 3 (49316972-51832015) drawn by FOCUS software; **(B)** shows the most significant gene PIM3 in the region of chromosome 22 (49824534-51243298) drawn by FOCUS software.

We used FOCUS software fine-grained mapping of TWAS associations to analyze the data of single european ancestral population. A total of 17 positive genes were obtained from whole blood tissue (Supplementary Table S9) under the screening conditions of *P*_FDR_ < 0.05 and PIP >0.8. FOCUS was able to generate a plot for each region containing predicted expression correlations. The TWAS summary statistics and PIP for each gene and the results were shown in Figure 4. In the validation dataset, 19 positive genes were obtained under the same selection criteria (Supplementary Table S10).

### 3.5 Intersection and MR results

**FIGURE 5:**
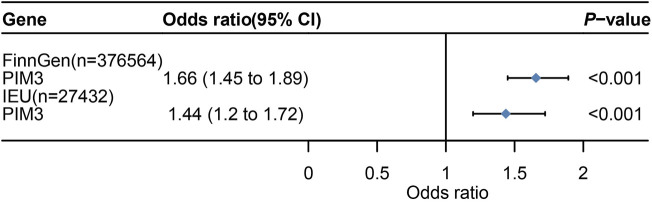
Mendelian randomization (MR) result. 95%CI,95% Confidence Interval. Figure 5 shows the Mendelian randomization (MR) results with PIM3 as the exposure and UC as the outcome.

Venn diagram displayed nominally significant genes obtained by the four analyses (Supplementary Figure S3). PIM3 was the significant genes after taking the intersection of the four analyses (Supplementary Table S11). MR analysis was conducted to screen qualified SNPs, and a total of 75 eligible IVs were included in subsequent analyses (Supplementary Table S12) (*P* < 5E-08). These 75 IVs clustered over a distance of 10,000 kb with an LD of r^2^ < 0.3 and were harmonized in both exposure and outcome GWAS datasets. A total of 2 IVs met the criteria (rs28645887, rs62231924). Therefore, MR analysis was performed using the IVW method, and 2 SNPs were included as IVs (*p* < 0.001,95%CI: 1.45-1.89) (Figure 5). In the validation dataset, the intersection of positive results from the four analyses also identified the same risk gene PIM3 (Supplementary Figure S4). IVs were the same and the MR analysis also applied the IVW method (*p* < 0.001, 95%CI: 1.20-1.72) (Figure 5).

### 3.6 Bayesian colocalization results

Bayesian colocalization analysis was performed on PIM3. The windows for colocalization analysis were set to 10 kb (Leclerc et al., 2018), and the result of PPH4 was 0.997 (Table 3). In the validation dataset, windows for colocalization analysis were set to the same parameters and the result for PPH4 was 0.953 (Table 4).

**TABLE 3:** Results of Bayesian colocalization analysis.

Gene	PPH0	PPH1	PPH2	PPH3	PPH4
PIM3	1.36E-26	2.31E-08	2.4E-21	3.10E-03	9.97E-01

**TABLE 4:** Results of Bayesian colocalization analysis [validation dataset (ieu-a-32)].

Gene	PPH0	PPH1	PPH2	PPH3	PPH4
PIM3	2.39E-20	4.11E-02	3.96E-21	5.84E-03	9.53E-01

## 4 Discussion

Based on the GWAS datasets of UC, we systematically evaluated genetically predicted associations between gene expression and UC risk. MAGMA was used to annotate UC risk genes, and then UTMOST, FUSION and conditional and joint analysis were used to identify UC risk genes. To further improve the accuracy of the results, FOCUS was used to fine-map the UC risk genes. PIM3 was the common gene after taking the intersection of four genetic analysis methods (MAGMA, UTMOST, FUSION and FOCUS). Finally, MR and Bayesian colocalization analyses were performed on PIM3 to determine the obvious causal relationship between the gene and UC. This result was verified using the GWAS dataset of UC from the IEU. The current findings could improve our understanding on the genetic inheritance and etiology of UC.

Previous research results showed that the cross-tissue TWAS analysis method can effectively obtain more significant risk genes (Zhu et al., 2021). Our research is innovative as currently there is no study performed to identify risk genes for UC using cross-tissue TWAS analysis. The eQTL data used in this study was the GTExv8 version. With the expansion of GTEx project data, our research results could be more stable and accurate.

PIM kinases (Provirus Integration site for Moloney leukemia virus) are a family of serine/threonine protein kinases that play important roles in cell development, immune regulation, and tumorigenesis (Bellon and Nicot, 2023). As the third member of this kinase family, PIM3 could catalyze histone phosphorylation and autophosphorylation (Feldman et al., 1998). Previous studies considered PIM3 as a gene closely related to the occurrence of various cancers such as colon cancer, liver cancer, pancreatic cancer, prostate cancer, gastric cancer, and breast cancer (Brault et al., 2010; Du et al., 2015; Qi et al., 2019; Wang et al., 2019; Marayati et al., 2022). Our study found that PIM3 was also highly correlated with the risk of UC. Evidence also showed that that the incidence of colorectal cancer in UC patients is approximately 2–3 times higher than that in the normal population (Shah and Itzkowitz, 2022). Chronic inflammation is a driving factor in tumor progression, and PIM3 is significantly overexpressed in UC-related colorectal cancer (Zhou et al., 2021). Hence, our study provided a gene with the potential to predict the progression from UC to colorectal cancer.

The PIM kinase family plays a crucial role in the inflammatory process. Studies have shown that the three PIM family kinases also fulfil broader pathological functions in cardiovascular diseases, including in inflammation, thrombosis and cardiac injury (Nock et al., 2023). In a transcriptomic study of inflammation in psoriasis, PIM3 kinase is located among key regulator transcripts (Garshick et al., 2023). Similarly, another study on chronic obstructive pulmonary disease (COPD) showed that the mRNA and protein levels of PIM3 were upregulated in COPD tissue as compared to normal lung tissue, which was also verified using animal experiments. Lung damage in COPD patients could be improved through inhibiting the expression of PIM3 (Yang et al., 2017). For UC, animal experiments have shown that PIM1 and PIM3 play an important regulatory role in the differentiation and proliferation of CD4^+^ T cells, and that a higher activity of these two kinases may help sustain the disease severity (Jackson et al., 2012). It has been reported that PIM3 could regulate the downstream of the JAK/STAT pathway, and that the gene is upregulated in Th17 cells via the IL6/STAT3 axis (Buchacher et al., 2023). IL6 can induce the expression of PIM3 in the experiment, and a lack of STAT3 downregulates all the three PIMs. These findings suggested a strong potential of studying PIM3 in the pathogenesis of inflammatory diseases.

PIM3 has also been found to be associated with ferroptosis, which is a recently discovered mode of cell death distinct from apoptosis and autophagy. Ferroptosis is characterized by the accumulation of iron-dependent lipid peroxidation to lethal levels (Dixon et al., 2012; Stockwell et al., 2017; Galluzzi et al., 2018). Our knowledge on the role of ferroptosis has been extended from tumors to gastrointestinal diseases (Song et al., 2019; Xu et al., 2020). Researches have shown that ferroptosis is abnormally active in UC patients, and that blocking the ferroptosis process can effectively alleviate UC symptoms and promote the repair of the intestinal mucosal barrier (Chen et al., 2020; Xu et al., 2020). The basic features of ferroptosis are the accumulation of lipid peroxidation, iron deposition, GPX4 inactivation, GSH depletion, among which the most significant characteristic is the substantial increase in reactive oxygen species (ROS). Noticeably, all these features are closely associated with the risk of UC (Wang et al., 2020; Chen et al., 2021; Tang et al., 2021). Li et al. (2024) validated the relationship between PIM3 and ferroptosis using a rat model of myocardial ischemia/reperfusion (I/R) injury and a cell model induced by oxygen-glucose deprivation/reoxygenation (OGD/R). Their findings showed that myocardial I/R modeling or OGD/R treatment can upregulate the expression of PIM3, which in turn promotes the levels of ferroptosis, evidenced by increased ROS and iron content as well as downregulated SOD and GPX4. Silencing PIM3 can suppress ferroptosis and reduce ROS levels, thereby improving myocardial model injury and promoting cell survival rates. Based on these results, we can reasonably speculate that there was a close relationship between PIM3 and ferroptosis, and that ferroptosis was related to the onset of UC. This could be a potential direction for expanding our understanding on the pathogenesis of UC and treating UC.

However, some limitations in this study should be noted. Firstly, due to the criteria for selecting significant cis-heritable genes in TWAS analysis, genes were not all included in the study and some UC-related SNPs but were irrelevant to the cis-expression were not considered. Secondly, the race of our GWAS data and the reference GTExv8 version eQTL data were European population, which may have certain impact on applying the current results to other races. Thirdly, with the continuous release of high-throughput data from more tissues and UC GWAS datasets from diverse ancestral populations, cross-tissue correlation analysis combined with other GWAS analysis strategies is expected to show stronger statistical power and provide deeper insights into UC genetics. Finally, the underlying mechanism of PIM3 has not been verified using experiments.

## 5 Conclusion

In summary, we discovered PIM3 as a key risk gene for UC applying four genetic analyses, providing novel insights into the underlying genetic structure of UC. However, further experimental studies are still needed to elucidate the mechanism of action of PIM3 in UC.

## Data Availability

The original contributions presented in the study are included in the article/Supplementary Material, further inquiries can be directed to the corresponding author.
