# Supplementary material for: Gaining new insights into the etiology of ulcerative colitis through a cross-tissue transcriptome-wide association study
Source: Front Genet. 2024 Jul 18;15:1425370. doi: 10.3389/fgene.2024.1425370 (PMC11291327; doi:10.3389/fgene.2024.1425370)

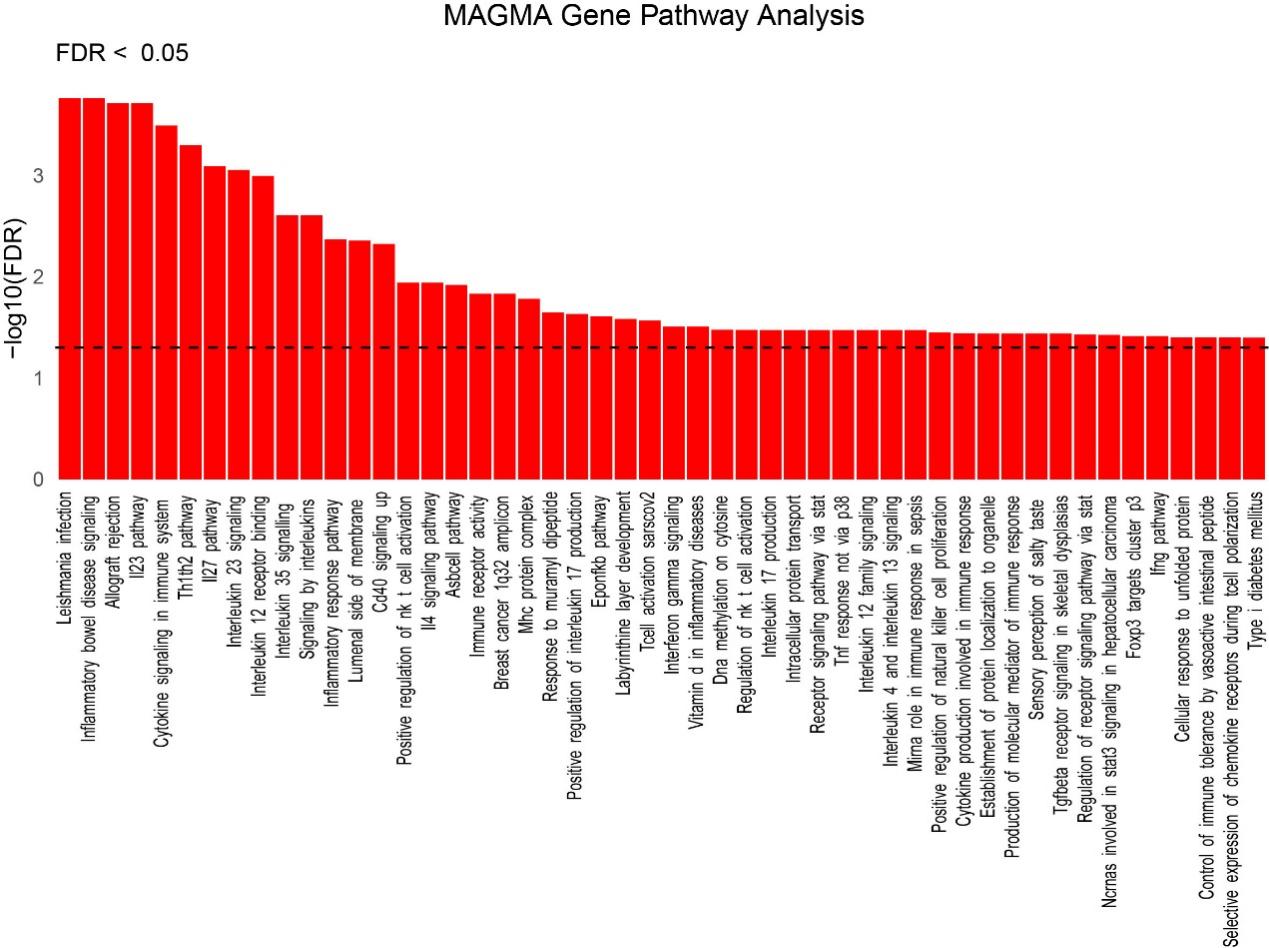
Figure S1 Pathway enrichment results of MAGMA ((validation dataset (ieu-a-32))

Figure S2 tissue-specific enrichment results of MAGMA ((validation dataset (ieu-a-32))


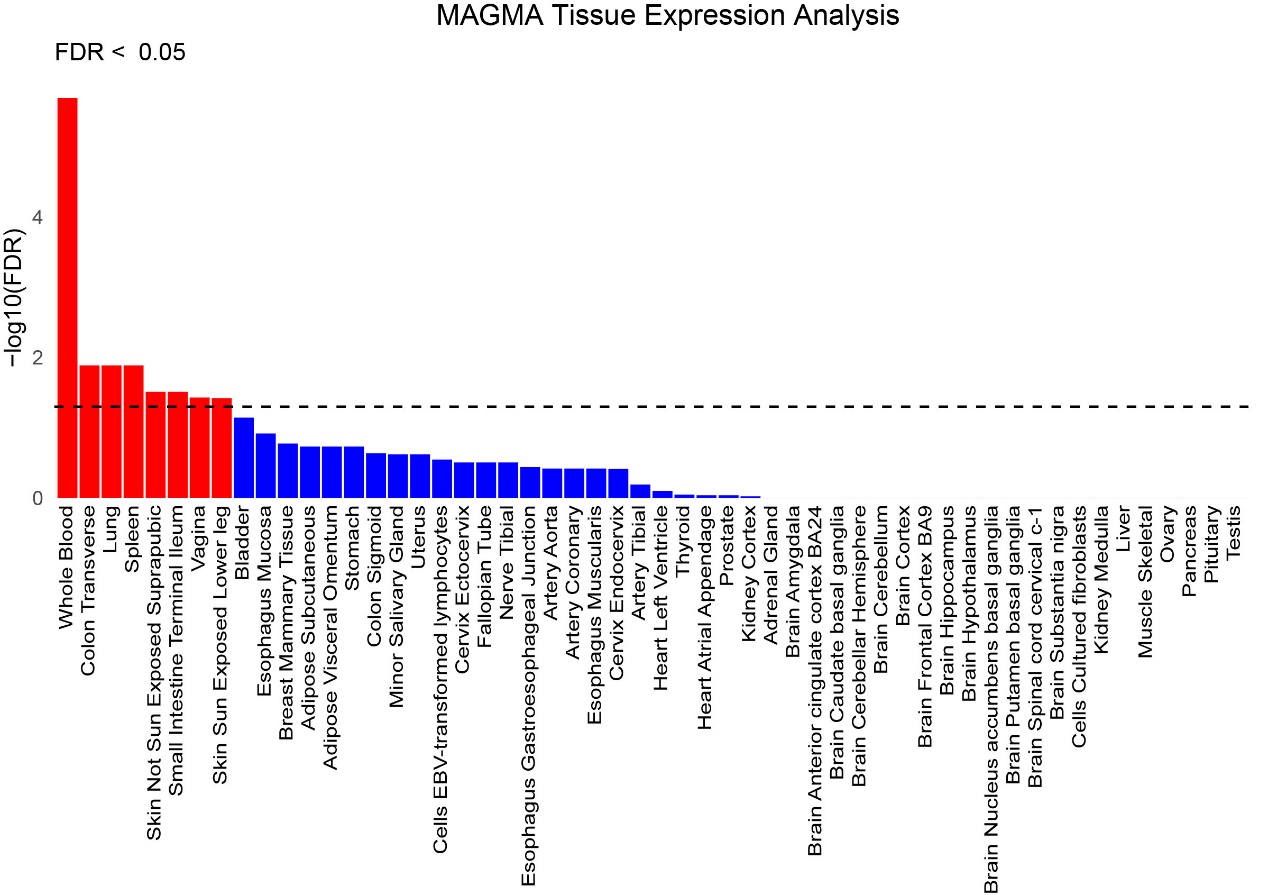


Figure S3 Venn Diagram of Intersection of Results from Four Analytical Methods


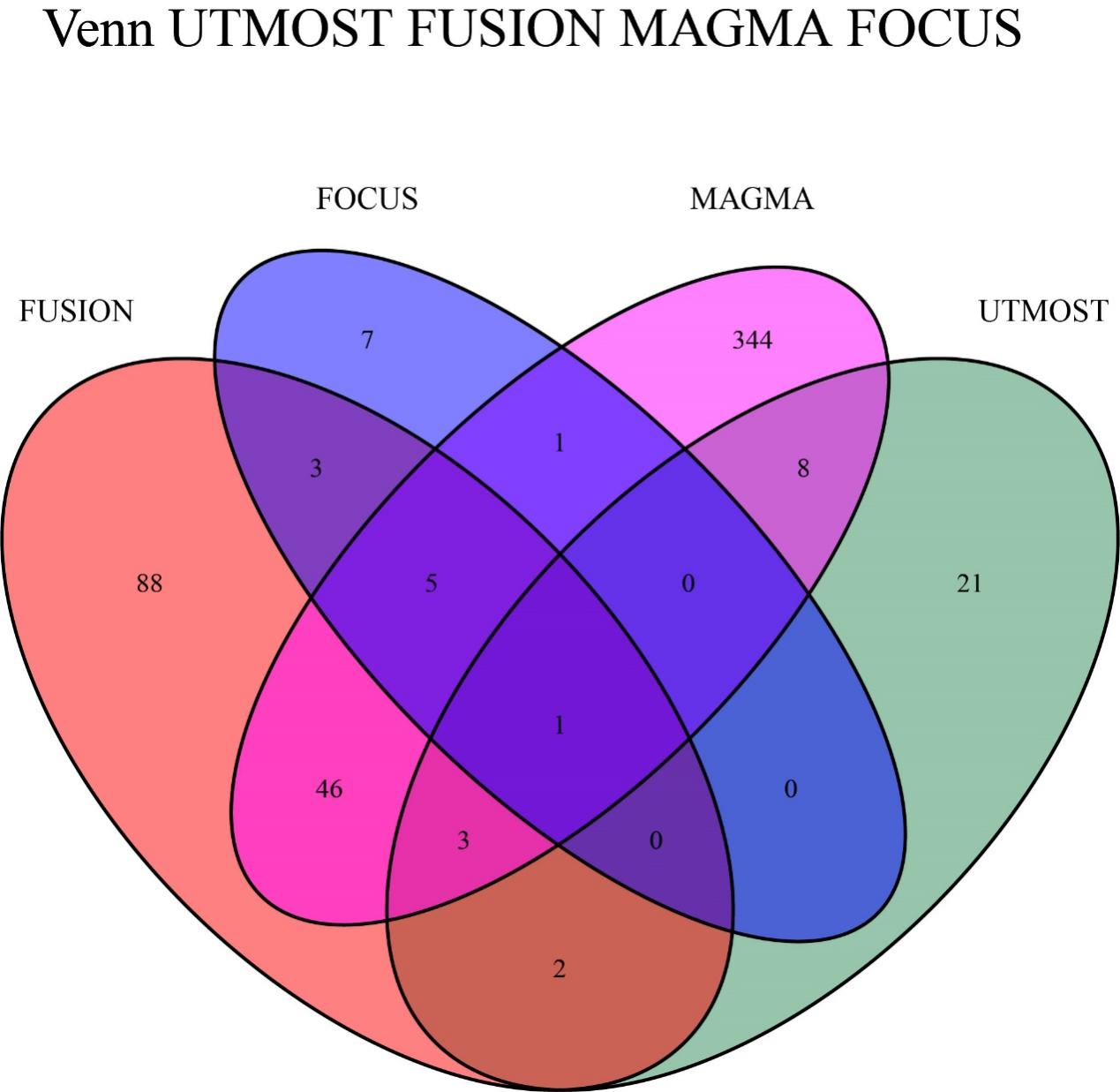


Figure S4 Venn Diagram of Intersection of Results from Four Analytical Methods ((validation dataset (ieu-a-32))


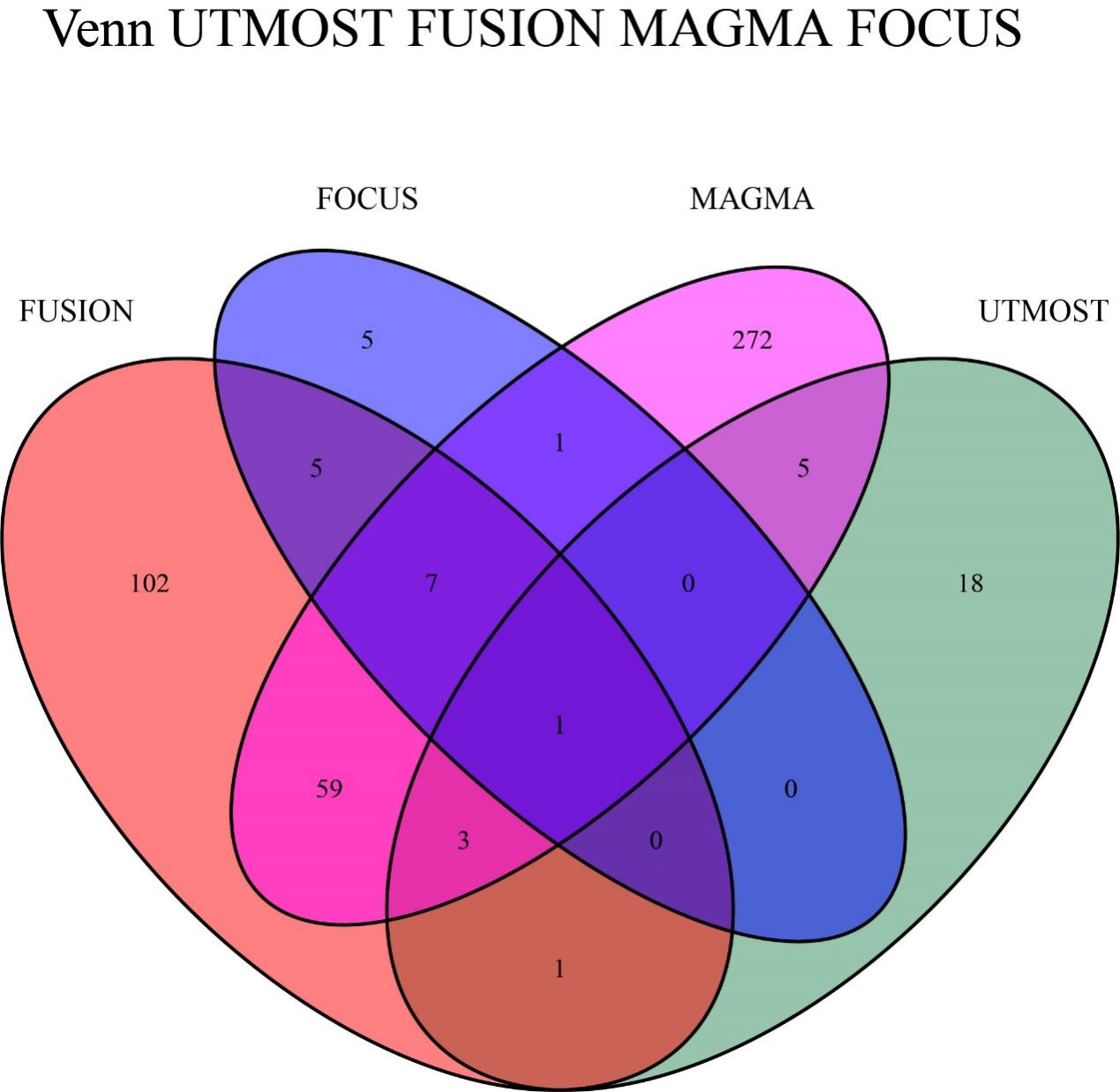

Supplement: Supplementary file 1 [file Table1.DOCX]
