# Supplementary material for: Gaining new insights into the etiology of ulcerative colitis through a cross-tissue transcriptome-wide association study
Source: Front Genet. 2024 Jul 18;15:1425370. doi: 10.3389/fgene.2024.1425370 (PMC11291327; doi:10.3389/fgene.2024.1425370)
Supplement: Supplementary file 2 [file Table2.DOCX]

**Table S1 UC risk genes identified through gene-based association studies**

| **Gene** | **CHR** | | **N** | **ZSTAT** | ***P*** | ***P*_FDR_** |
| --- | --- | --- | --- | --- | --- | --- |
| AMT | 3 | 376564 | | 8.4354 | 1.65E-17 | 1.89E-13 |
| TNRC18 | 7 | 376564 | | 8.4135 | 1.99E-17 | 1.89E-13 |
| NICN1 | 3 | 376564 | | 8.0878 | 3.04E-16 | 1.92E-12 |
| APEH | 3 | 376564 | | 8.0156 | 5.48E-16 | 2.00E-12 |
| MST1 | 3 | 376564 | | 8.01 | 5.74E-16 | 2.00E-12 |
| BSN | 3 | 376564 | | 7.9981 | 6.32E-16 | 2.00E-12 |
| RHOA | 3 | 376564 | | 7.8287 | 2.46E-15 | 6.69E-12 |
| TCTA | 3 | 376564 | | 7.6941 | 7.12E-15 | 1.69E-11 |
| HLA-DRA | 6 | 376564 | | 7.5609 | 2.00E-14 | 4.23E-11 |
| HLA-DQB1 | 6 | 376564 | | 7.4497 | 4.68E-14 | 8.89E-11 |
| OTUD3 | 1 | 376564 | | 7.3906 | 7.31E-14 | 1.26E-10 |
| HLA-DQA1 | 6 | 376564 | | 7.3021 | 1.42E-13 | 2.24E-10 |
| DAG1 | 3 | 376564 | | 7.2194 | 2.61E-13 | 3.82E-10 |
| SLC29A4 | 7 | 376564 | | 7.1555 | 4.17E-13 | 5.66E-10 |
| DLD | 7 | 376564 | | 7.0772 | 7.35E-13 | 9.32E-10 |
| MMD2 | 7 | 376564 | | 6.9264 | 2.16E-12 | 2.57E-09 |
| RNF123 | 3 | 376564 | | 6.8436 | 3.86E-12 | 4.32E-09 |
| FBXL18 | 7 | 376564 | | 6.7667 | 6.59E-12 | 6.96E-09 |
| IL17REL | 22 | 376564 | | 6.6916 | 1.10E-11 | 1.10E-08 |
| FCGR2A | 1 | 376564 | | 6.5997 | 2.06E-11 | 1.96E-08 |
| PIM3 | 22 | 376564 | | 6.5464 | 2.95E-11 | 2.67E-08 |
| ZPBP2 | 17 | 376564 | | 6.5246 | 3.41E-11 | 2.95E-08 |
| GPR35 | 2 | 376564 | | 6.4515 | 5.54E-11 | 4.58E-08 |
| IL10 | 1 | 376564 | | 6.3873 | 8.44E-11 | 6.69E-08 |
| IL23R | 1 | 376564 | | 6.3361 | 1.18E-10 | 8.96E-08 |
| LAMB1 | 7 | 376564 | | 6.3259 | 1.26E-10 | 9.20E-08 |
| CDHR4 | 3 | 376564 | | 6.292 | 1.57E-10 | 1.10E-07 |
| DXO | 6 | 376564 | | 6.2755 | 1.74E-10 | 1.18E-07 |
| TRAIP | 3 | 376564 | | 6.2054 | 2.73E-10 | 1.79E-07 |
| C1orf106 | 1 | 376564 | | 6.1094 | 5.00E-10 | 2.97E-07 |
| IP6K1 | 3 | 376564 | | 6.1094 | 5.00E-10 | 2.97E-07 |
| GPX1 | 3 | 376564 | | 6.1094 | 5.00E-10 | 2.97E-07 |
| NKX2-3 | 10 | 376564 | | 6.0808 | 5.98E-10 | 3.45E-07 |
| HELZ2 | 20 | 376564 | | 6.0027 | 9.71E-10 | 5.43E-07 |
| GSDMB | 17 | 376564 | | 5.8277 | 2.81E-09 | 1.53E-06 |
| ORMDL3 | 17 | 376564 | | 5.7847 | 3.63E-09 | 1.92E-06 |
| IRF1 | 5 | 376564 | | 5.7475 | 4.53E-09 | 2.33E-06 |
| RTEL1-TNFRSF6B | 20 | 376564 | | 5.7253 | 5.16E-09 | 2.58E-06 |
| RTEL1 | 20 | 376564 | | 5.6514 | 7.96E-09 | 3.88E-06 |
| USP4 | 3 | 376564 | | 5.4909 | 2.00E-08 | 9.51E-06 |
| GSDMA | 17 | 376564 | | 5.3945 | 3.44E-08 | 1.59E-05 |
| TNFSF15 | 9 | 376564 | | 5.3541 | 4.30E-08 | 1.95E-05 |
| RPP21 | 6 | 376564 | | 5.3417 | 4.60E-08 | 2.04E-05 |
| JAK2 | 9 | 376564 | | 5.331 | 4.88E-08 | 2.11E-05 |
| PLA2G2E | 1 | 376564 | | 5.3273 | 4.98E-08 | 2.11E-05 |
| PUS10 | 2 | 376564 | | 5.3061 | 5.60E-08 | 2.31E-05 |
| AL161450.1 | 9 | 376564 | | 5.2773 | 6.55E-08 | 2.64E-05 |
| HLA-F | 6 | 376564 | | 5.274 | 6.67E-08 | 2.64E-05 |
| LPP | 3 | 376564 | | 5.2398 | 8.04E-08 | 3.12E-05 |
| FADS2 | 11 | 376564 | | 5.2187 | 9.01E-08 | 3.43E-05 |
| CCHCR1 | 6 | 376564 | | 5.2034 | 9.79E-08 | 3.65E-05 |
| MAPKAPK2 | 1 | 376564 | | 5.1835 | 1.09E-07 | 3.98E-05 |
| ERRFI1 | 1 | 376564 | | 5.1767 | 1.13E-07 | 4.05E-05 |
| C5orf56 | 5 | 376564 | | 5.1288 | 1.46E-07 | 5.13E-05 |
| VARS | 6 | 376564 | | 5.1073 | 1.63E-07 | 5.65E-05 |
| CUEDC2 | 10 | 376564 | | 5.0967 | 1.73E-07 | 5.87E-05 |
| PNPLA1 | 6 | 376564 | | 5.0848 | 1.84E-07 | 6.14E-05 |
| FCGR3A | 1 | 376564 | | 5.075 | 1.94E-07 | 6.35E-05 |
| KIF21B | 1 | 376564 | | 5.048 | 2.23E-07 | 7.19E-05 |
| STK19 | 6 | 376564 | | 5.0047 | 2.80E-07 | 8.86E-05 |
| CXCR2 | 2 | 376564 | | 4.9748 | 3.27E-07 | 0.000101784 |
| KRT222 | 17 | 376564 | | 4.9216 | 4.29E-07 | 0.000131581 |
| PARK7 | 1 | 376564 | | 4.8671 | 5.66E-07 | 0.000168847 |
| HLA-DMA | 6 | 376564 | | 4.8648 | 5.73E-07 | 0.000168847 |
| HSPA6 | 1 | 376564 | | 4.8633 | 5.77E-07 | 0.000168847 |
| NFKB2 | 10 | 376564 | | 4.8243 | 7.03E-07 | 0.000202355 |
| KRT222 | 17 | 376564 | | 4.8172 | 7.28E-07 | 0.000206567 |
| TNXB | 6 | 376564 | | 4.8081 | 7.62E-07 | 0.000212954 |
| CCZ1 | 7 | 376564 | | 4.8024 | 7.84E-07 | 0.000215926 |
| C3orf62 | 3 | 376564 | | 4.7297 | 1.12E-06 | 0.000300506 |
| TNFRSF6B | 20 | 376564 | | 4.7283 | 1.13E-06 | 0.000300506 |
| MST1R | 3 | 376564 | | 4.7272 | 1.14E-06 | 0.000300506 |
| TCF19 | 6 | 376564 | | 4.7198 | 1.18E-06 | 0.000303328 |
| HLA-DOA | 6 | 376564 | | 4.7197 | 1.18E-06 | 0.000303328 |
| POU5F1 | 6 | 376564 | | 4.7073 | 1.26E-06 | 0.000318117 |
| PNKD | 2 | 376564 | | 4.7022 | 1.29E-06 | 0.000321088 |
| MIEN1 | 17 | 376564 | | 4.7001 | 1.30E-06 | 0.000321088 |
| CCRL2 | 3 | 376564 | | 4.6944 | 1.34E-06 | 0.000325917 |
| CACNA1S | 1 | 376564 | | 4.6782 | 1.45E-06 | 0.000348286 |
| TRIM31 | 6 | 376564 | | 4.6733 | 1.48E-06 | 0.00035225 |
| IP6K2 | 3 | 376564 | | 4.6641 | 1.55E-06 | 0.000363673 |
| ATAD3A | 1 | 376564 | | 4.636 | 1.78E-06 | 0.000408943 |
| TMBIM1 | 2 | 376564 | | 4.6349 | 1.79E-06 | 0.000408943 |
| ATF6B | 6 | 376564 | | 4.6245 | 1.88E-06 | 0.000420076 |
| OR2H2 | 6 | 376564 | | 4.6244 | 1.88E-06 | 0.000420076 |
| C3orf84 | 3 | 376564 | | 4.6153 | 1.96E-06 | 0.000433915 |
| ITPR3 | 6 | 376564 | | 4.61 | 2.01E-06 | 0.00044005 |
| NELFE | 6 | 376564 | | 4.5883 | 2.23E-06 | 0.000474261 |
| CCDC36 | 3 | 376564 | | 4.5878 | 2.24E-06 | 0.000474261 |
| AAMP | 2 | 376564 | | 4.5873 | 2.25E-06 | 0.000474261 |
| MON1A | 3 | 376564 | | 4.5844 | 2.28E-06 | 0.000475588 |
| IRF5 | 7 | 376564 | | 4.5766 | 2.36E-06 | 0.000488252 |
| AGER | 6 | 376564 | | 4.5742 | 2.39E-06 | 0.000488501 |
| RGS19 | 20 | 376564 | | 4.5667 | 2.48E-06 | 0.000501142 |
| ACOXL | 2 | 376564 | | 4.5509 | 2.67E-06 | 0.000534389 |
| TMCO4 | 1 | 376564 | | 4.5144 | 3.18E-06 | 0.000628848 |
| ZGPAT | 20 | 376564 | | 4.4994 | 3.41E-06 | 0.000662883 |
| SMARCE1 | 17 | 376564 | | 4.4988 | 3.42E-06 | 0.000662883 |
| RP4-583P15.15 | 20 | 376564 | | 4.4797 | 3.74E-06 | 0.000717809 |
| CAMSAP2 | 1 | 376564 | | 4.4655 | 3.99E-06 | 0.000759337 |
| KLHDC8B | 3 | 376564 | | 4.4525 | 4.24E-06 | 0.000794138 |
| FADS1 | 11 | 376564 | | 4.4516 | 4.26E-06 | 0.000794138 |
| RTP3 | 3 | 376564 | | 4.4439 | 4.42E-06 | 0.00081524 |
| ARFRP1 | 20 | 376564 | | 4.4306 | 4.70E-06 | 0.000858785 |
| MED24 | 17 | 376564 | | 4.4182 | 4.98E-06 | 0.000899866 |
| BRD2 | 6 | 376564 | | 4.4164 | 5.02E-06 | 0.000899866 |
| KRT28 | 17 | 376564 | | 4.3868 | 5.75E-06 | 0.001021815 |
| TMEM99 | 17 | 376564 | | 4.3637 | 6.39E-06 | 0.001125627 |
| UBA7 | 3 | 376564 | | 4.359 | 6.53E-06 | 0.001130256 |
| EHMT2 | 6 | 376564 | | 4.3588 | 6.54E-06 | 0.001130256 |
| GMEB2 | 20 | 376564 | | 4.3446 | 6.98E-06 | 0.001194833 |
| SLC2A4RG | 20 | 376564 | | 4.3283 | 7.51E-06 | 0.001275587 |
| DPCR1 | 6 | 376564 | | 4.3252 | 7.62E-06 | 0.001282149 |
| CYTH3 | 7 | 376564 | | 4.3117 | 8.10E-06 | 0.001350898 |
| DYRK3 | 1 | 376564 | | 4.2973 | 8.64E-06 | 0.001428999 |
| TAP2 | 6 | 376564 | | 4.2721 | 9.68E-06 | 0.001586632 |
| IKZF3 | 17 | 376564 | | 4.2631 | 1.01E-05 | 0.001638196 |
| SKIV2L | 6 | 376564 | | 4.239 | 1.12E-05 | 0.001808784 |
| CD28 | 2 | 376564 | | 4.2358 | 1.14E-05 | 0.001818826 |
| ACTB | 7 | 376564 | | 4.2336 | 1.15E-05 | 0.001821571 |
| CAMKV | 3 | 376564 | | 4.2003 | 1.33E-05 | 0.002094195 |
| KRI1 | 19 | 376564 | | 4.1944 | 1.37E-05 | 0.002131258 |
| TRIM39-RPP21 | 6 | 376564 | | 4.1618 | 1.58E-05 | 0.002440054 |
| OCM | 7 | 376564 | | 4.1448 | 1.70E-05 | 0.002607727 |
| TMEM258 | 11 | 376564 | | 4.1104 | 1.97E-05 | 0.002998199 |
| CTD-2330K9.3 | 3 | 376564 | | 4.1077 | 2.00E-05 | 0.002998199 |
| MGAT2 | 14 | 376564 | | 4.1071 | 2.00E-05 | 0.002998199 |
| KRT24 | 17 | 376564 | | 4.0965 | 2.10E-05 | 0.003101888 |
| PSMD3 | 17 | 376564 | | 4.0957 | 2.10E-05 | 0.003101888 |
| POM121L2 | 6 | 376564 | | 4.0923 | 2.14E-05 | 0.003123215 |
| C6orf10 | 6 | 376564 | | 4.087 | 2.18E-05 | 0.003170774 |
| LIME1 | 20 | 376564 | | 4.082 | 2.23E-05 | 0.003216028 |
| HIST1H4H | 6 | 376564 | | 4.0766 | 2.29E-05 | 0.003237751 |
| GGNBP1 | 6 | 376564 | | 4.076 | 2.29E-05 | 0.003237751 |
| TMEM163 | 2 | 376564 | | 4.0713 | 2.34E-05 | 0.003237751 |
| RSPH10B | 7 | 376564 | | 4.0707 | 2.34E-05 | 0.003237751 |
| NOTCH4 | 6 | 376564 | | 4.0705 | 2.35E-05 | 0.003237751 |
| ELOVL3 | 10 | 376564 | | 4.0692 | 2.36E-05 | 0.003237751 |
| STMN3 | 20 | 376564 | | 4.0684 | 2.37E-05 | 0.003237751 |
| QRICH1 | 3 | 376564 | | 4.0598 | 2.46E-05 | 0.003335208 |
| IMPDH2 | 3 | 376564 | | 4.0564 | 2.49E-05 | 0.003344865 |
| GPC2 | 7 | 376564 | | 4.0558 | 2.50E-05 | 0.003344865 |
| IL19 | 1 | 376564 | | 4.0468 | 2.60E-05 | 0.003451892 |
| PDZD7 | 10 | 376564 | | 4.0442 | 2.63E-05 | 0.003465547 |
| NFATC3 | 16 | 376564 | | 4.0387 | 2.69E-05 | 0.003515733 |
| TRIM40 | 6 | 376564 | | 4.0376 | 2.70E-05 | 0.003515733 |
| DDR1 | 6 | 376564 | | 4.0317 | 2.77E-05 | 0.003526034 |
| CHFR | 12 | 376564 | | 4.0314 | 2.77E-05 | 0.003526034 |
| KRT10 | 17 | 376564 | | 4.0311 | 2.78E-05 | 0.003526034 |
| C6orf48 | 6 | 376564 | | 4.0306 | 2.78E-05 | 0.003526034 |
| PGAP3 | 17 | 376564 | | 4.0227 | 2.88E-05 | 0.003622288 |
| DUS2 | 16 | 376564 | | 4.0161 | 2.96E-05 | 0.003700641 |
| SH3BGR | 21 | 376564 | | 4.0088 | 3.05E-05 | 0.003776443 |
| IRGM | 5 | 376564 | | 4.0075 | 3.07E-05 | 0.003776443 |
| C3orf20 | 3 | 376564 | | 4.0067 | 3.08E-05 | 0.003776443 |
| SAPCD1 | 6 | 376564 | | 4.0035 | 3.12E-05 | 0.003803541 |
| RADIL | 7 | 376564 | | 3.9888 | 3.32E-05 | 0.004020161 |
| PMS2 | 7 | 376564 | | 3.9694 | 3.60E-05 | 0.004335471 |
| DALRD3 | 3 | 376564 | | 3.9651 | 3.67E-05 | 0.004346347 |
| P4HA2 | 5 | 376564 | | 3.9628 | 3.70E-05 | 0.004346347 |
| ZSCAN23 | 6 | 376564 | | 3.9627 | 3.71E-05 | 0.004346347 |
| IRF6 | 1 | 376564 | | 3.9601 | 3.75E-05 | 0.004346347 |
| CSRP2 | 12 | 376564 | | 3.9584 | 3.77E-05 | 0.004346347 |
| CFB | 6 | 376564 | | 3.9569 | 3.80E-05 | 0.004346347 |
| GABBR1 | 6 | 376564 | | 3.9566 | 3.80E-05 | 0.004346347 |
| CFB | 6 | 376564 | | 3.9565 | 3.80E-05 | 0.004346347 |
| C20orf112 | 20 | 376564 | | 3.9555 | 3.82E-05 | 0.004346347 |
| ZNRF2 | 7 | 376564 | | 3.9528 | 3.86E-05 | 0.004370606 |
| CCNL2 | 1 | 376564 | | 3.9488 | 3.93E-05 | 0.004417751 |
| TAP2 | 6 | 376564 | | 3.9448 | 3.99E-05 | 0.004466243 |
| C1orf141 | 1 | 376564 | | 3.9356 | 4.15E-05 | 0.004612891 |
| GPR183 | 13 | 376564 | | 3.9295 | 4.26E-05 | 0.004704338 |
| HSD17B8 | 6 | 376564 | | 3.9214 | 4.40E-05 | 0.004836442 |
| GTF2H4 | 6 | 376564 | | 3.92 | 4.43E-05 | 0.004836442 |
| NCKIPSD | 3 | 376564 | | 3.907 | 4.67E-05 | 0.005040278 |
| ARPC2 | 2 | 376564 | | 3.9069 | 4.67E-05 | 0.005040278 |
| INSL6 | 9 | 376564 | | 3.906 | 4.69E-05 | 0.005040278 |
| VWA1 | 1 | 376564 | | 3.9 | 4.81E-05 | 0.005136281 |
| STAT3 | 17 | 376564 | | 3.8973 | 4.86E-05 | 0.005166 |
| ATG4D | 19 | 376564 | | 3.8929 | 4.95E-05 | 0.005231194 |
| LST1 | 6 | 376564 | | 3.8859 | 5.10E-05 | 0.00535438 |
| ATAD3B | 1 | 376564 | | 3.8755 | 5.32E-05 | 0.005558315 |
| CELSR3 | 3 | 376564 | | 3.8679 | 5.49E-05 | 0.005667744 |
| PRRT1 | 6 | 376564 | | 3.8673 | 5.50E-05 | 0.005667744 |
| NEURL1 | 10 | 376564 | | 3.8667 | 5.52E-05 | 0.005667744 |
| HLA-B | 6 | 376564 | | 3.8592 | 5.69E-05 | 0.005814198 |
| PNMT | 17 | 376564 | | 3.8575 | 5.73E-05 | 0.005823872 |
| SEPT1 | 16 | 376564 | | 3.8499 | 5.91E-05 | 0.005973701 |
| C2 | 6 | 376564 | | 3.8473 | 5.97E-05 | 0.006005766 |
| RP4-583P15.14 | 20 | 376564 | | 3.8431 | 6.07E-05 | 0.006062736 |
| HIST1H2BK | 6 | 376564 | | 3.8417 | 6.11E-05 | 0.006062736 |
| SSU72 | 1 | 376564 | | 3.841 | 6.13E-05 | 0.006062736 |
| ETV7 | 6 | 376564 | | 3.8399 | 6.15E-05 | 0.006062736 |
| MXRA8 | 1 | 376564 | | 3.8379 | 6.20E-05 | 0.006072948 |
| RBAK-RBAKDN | 7 | 376564 | | 3.8361 | 6.25E-05 | 0.006072948 |
| SYNGR1 | 22 | 376564 | | 3.8357 | 6.26E-05 | 0.006072948 |
| RXRB | 6 | 376564 | | 3.8272 | 6.48E-05 | 0.006252496 |
| OPRL1 | 20 | 376564 | | 3.8248 | 6.54E-05 | 0.006282559 |
| AL645728.1 | 1 | 376564 | | 3.8184 | 6.72E-05 | 0.006400243 |
| TSTA3 | 8 | 376564 | | 3.8178 | 6.73E-05 | 0.006400243 |
| WIPI2 | 7 | 376564 | | 3.7973 | 7.31E-05 | 0.006912098 |
| TM9SF4 | 20 | 376564 | | 3.7963 | 7.34E-05 | 0.006912098 |
| ACMSD | 2 | 376564 | | 3.7856 | 7.67E-05 | 0.007179696 |
| CYP21A2 | 6 | 376564 | | 3.7741 | 8.03E-05 | 0.007482133 |
| ZNF48 | 16 | 376564 | | 3.7715 | 8.11E-05 | 0.007521527 |
| PTPN2 | 18 | 376564 | | 3.7704 | 8.15E-05 | 0.007521527 |
| REL | 2 | 376564 | | 3.7614 | 8.45E-05 | 0.007759519 |
| SEPT1 | 16 | 376564 | | 3.759 | 8.53E-05 | 0.00779451 |
| RBBP8 | 18 | 376564 | | 3.7505 | 8.82E-05 | 0.008003993 |
| C9orf91 | 9 | 376564 | | 3.75 | 8.84E-05 | 0.008003993 |
| ITGAL | 16 | 376564 | | 3.7486 | 8.89E-05 | 0.008011109 |
| PTPRC | 1 | 376564 | | 3.7356 | 9.36E-05 | 0.008396764 |
| HCG27 | 6 | 376564 | | 3.7309 | 9.54E-05 | 0.008474642 |
| AL139099.1 | 14 | 376564 | | 3.7309 | 9.54E-05 | 0.008474642 |
| VARS2 | 6 | 376564 | | 3.7285 | 9.63E-05 | 0.00851516 |
| LSM2 | 6 | 376564 | | 3.726 | 9.73E-05 | 0.00852271 |
| UBAC2 | 13 | 376564 | | 3.726 | 9.73E-05 | 0.00852271 |
| PLA2G5 | 1 | 376564 | | 3.7112 | 0.00010312 | 0.008926568 |
| HLA-A | 6 | 376564 | | 3.7101 | 0.0001036 | 0.008926568 |
| NDUFV2 | 18 | 376564 | | 3.7097 | 0.00010376 | 0.008926568 |
| BTNL2 | 6 | 376564 | | 3.7096 | 0.00010377 | 0.008926568 |
| NKAPL | 6 | 376564 | | 3.7059 | 0.00010532 | 0.009019092 |
| RP4-758J18.2 | 1 | 376564 | | 3.6997 | 0.00010791 | 0.009199448 |
| RLTPR | 16 | 376564 | | 3.6945 | 0.00011015 | 0.009327219 |
| CPSF3L | 1 | 376564 | | 3.694 | 0.00011039 | 0.009327219 |
| ADRA2B | 2 | 376564 | | 3.6833 | 0.00011514 | 0.009685516 |
| DIEXF | 1 | 376564 | | 3.6678 | 0.00012234 | 0.01024584 |
| SLC25A15 | 13 | 376564 | | 3.6653 | 0.00012351 | 0.010288355 |
| TTLL8 | 22 | 376564 | | 3.6645 | 0.00012393 | 0.010288355 |
| HIST1H2BJ | 6 | 376564 | | 3.6581 | 0.00012704 | 0.010500685 |
| IL24 | 1 | 376564 | | 3.653 | 0.00012959 | 0.010665089 |
| GPX4 | 19 | 376564 | | 3.6507 | 0.00013076 | 0.010714993 |
| TRIM26 | 6 | 376564 | | 3.6467 | 0.00013284 | 0.010838718 |
| RPL3 | 22 | 376564 | | 3.6404 | 0.00013612 | 0.011058877 |
| MPP4 | 2 | 376564 | | 3.6348 | 0.00013911 | 0.011253703 |
| ELF1 | 13 | 376564 | | 3.6337 | 0.00013971 | 0.011254351 |
| PDLIM4 | 5 | 376564 | | 3.6257 | 0.00014411 | 0.011559811 |
| TRIM39 | 6 | 376564 | | 3.6232 | 0.00014547 | 0.011619875 |
| AIMP2 | 7 | 376564 | | 3.622 | 0.0001462 | 0.011629323 |
| HAVCR1 | 5 | 376564 | | 3.6134 | 0.0001511 | 0.011969009 |
| PRKRIR | 11 | 376564 | | 3.609 | 0.00015368 | 0.012122865 |
| CD93 | 20 | 376564 | | 3.6031 | 0.00015724 | 0.012352437 |
| HIST3H3 | 1 | 376564 | | 3.5964 | 0.00016134 | 0.012622365 |
| ATAD3C | 1 | 376564 | | 3.5949 | 0.00016224 | 0.012640757 |
| ANKRD65 | 1 | 376564 | | 3.5905 | 0.00016499 | 0.012802286 |
| AC005549.3 | 17 | 376564 | | 3.5895 | 0.00016566 | 0.012802286 |
| RP11-410N8.4 | 20 | 376564 | | 3.5868 | 0.00016735 | 0.01288053 |
| ACD | 16 | 376564 | | 3.5852 | 0.00016844 | 0.012912149 |
| ASXL1 | 20 | 376564 | | 3.5831 | 0.00016975 | 0.012936605 |
| ZMAT5 | 22 | 376564 | | 3.5826 | 0.00017012 | 0.012936605 |
| KIF3B | 20 | 376564 | | 3.5788 | 0.00017256 | 0.013051957 |
| RNF216 | 7 | 376564 | | 3.5782 | 0.00017301 | 0.013051957 |
| DVL1 | 1 | 376564 | | 3.5756 | 0.00017473 | 0.013129613 |
| WDR41 | 5 | 376564 | | 3.5713 | 0.0001776 | 0.013292731 |
| RPS29 | 14 | 376564 | | 3.5498 | 0.00019278 | 0.014372316 |
| TBC1D10B | 16 | 376564 | | 3.5479 | 0.00019415 | 0.014415812 |
| ZKSCAN3 | 6 | 376564 | | 3.5469 | 0.00019488 | 0.014415812 |
| MMP10 | 11 | 376564 | | 3.5419 | 0.00019866 | 0.01463847 |
| BICD2 | 9 | 376564 | | 3.5347 | 0.0002041 | 0.014981255 |
| PRKAR2A | 3 | 376564 | | 3.5295 | 0.00020814 | 0.015219037 |
| SLC26A3 | 7 | 376564 | | 3.5279 | 0.00020942 | 0.01525396 |
| CAMK2A | 5 | 376564 | | 3.5188 | 0.00021671 | 0.015724709 |
| C6orf47 | 6 | 376564 | | 3.5164 | 0.00021875 | 0.015787771 |
| ZNF771 | 16 | 376564 | | 3.5158 | 0.00021924 | 0.015787771 |
| PDE4A | 19 | 376564 | | 3.4993 | 0.00023321 | 0.016644949 |
| FAIM3 | 1 | 376564 | | 3.4989 | 0.00023362 | 0.016644949 |
| HOXA13 | 7 | 376564 | | 3.4987 | 0.00023377 | 0.016644949 |
| CORO1A | 16 | 376564 | | 3.4944 | 0.00023758 | 0.01685311 |
| CUL2 | 10 | 376564 | | 3.4862 | 0.000245 | 0.017269845 |
| SLC22A5 | 5 | 376564 | | 3.4852 | 0.00024591 | 0.017269845 |
| QARS | 3 | 376564 | | 3.4849 | 0.00024618 | 0.017269845 |
| C2orf62 | 2 | 376564 | | 3.4826 | 0.00024832 | 0.017347363 |
| COL11A2 | 6 | 376564 | | 3.4817 | 0.00024911 | 0.017347363 |
| CRTC3 | 15 | 376564 | | 3.468 | 0.00026219 | 0.018191584 |
| MUC22 | 6 | 376564 | | 3.4654 | 0.00026468 | 0.018297569 |
| PHYKPL | 5 | 376564 | | 3.4569 | 0.00027322 | 0.018786574 |
| ERBB2 | 17 | 376564 | | 3.4564 | 0.00027373 | 0.018786574 |
| FOXP1 | 3 | 376564 | | 3.4472 | 0.00028317 | 0.01936455 |
| MSH5-SAPCD1 | 6 | 376564 | | 3.4442 | 0.0002864 | 0.019515234 |
| MSH5 | 6 | 376564 | | 3.4373 | 0.00029377 | 0.019945934 |
| GMPPB | 3 | 376564 | | 3.4327 | 0.00029879 | 0.020142896 |
| AMIGO3 | 3 | 376564 | | 3.4327 | 0.00029879 | 0.020142896 |
| SUFU | 10 | 376564 | | 3.428 | 0.00030406 | 0.020425741 |
| SLC7A6OS | 16 | 376564 | | 3.4243 | 0.00030817 | 0.020604999 |
| PSD | 10 | 376564 | | 3.4233 | 0.00030928 | 0.020604999 |
| RORC | 1 | 376564 | | 3.4227 | 0.00030998 | 0.020604999 |
| PHC2 | 1 | 376564 | | 3.4179 | 0.00031558 | 0.020904151 |
| AC110619.2 | 2 | 376564 | | 3.4161 | 0.00031765 | 0.020968209 |
| CREM | 10 | 376564 | | 3.4102 | 0.00032452 | 0.021347577 |
| GBF1 | 10 | 376564 | | 3.4013 | 0.00033529 | 0.021979994 |
| COMMD7 | 20 | 376564 | | 3.3988 | 0.0003384 | 0.022107637 |
| PPT2-EGFL8 | 6 | 376564 | | 3.3969 | 0.00034071 | 0.022182321 |
| FANCC | 9 | 376564 | | 3.3948 | 0.00034337 | 0.022279205 |
| KIAA1841 | 2 | 376564 | | 3.3932 | 0.00034538 | 0.022333399 |
| ASCC2 | 22 | 376564 | | 3.3895 | 0.00035004 | 0.022546661 |
| ZDHHC17 | 12 | 376564 | | 3.3888 | 0.00035105 | 0.022546661 |
| OVGP1 | 1 | 376564 | | 3.385 | 0.0003559 | 0.022781195 |
| EMC4 | 15 | 376564 | | 3.3811 | 0.00036102 | 0.02303138 |
| TMEM180 | 10 | 376564 | | 3.3769 | 0.00036649 | 0.023302145 |
| TNS4 | 17 | 376564 | | 3.3755 | 0.00036837 | 0.023340582 |
| PDE8B | 5 | 376564 | | 3.3746 | 0.00036955 | 0.023340582 |
| CABP7 | 22 | 376564 | | 3.3643 | 0.00038373 | 0.024020148 |
| VSX1 | 20 | 376564 | | 3.3642 | 0.00038378 | 0.024020148 |
| NOS3 | 7 | 376564 | | 3.364 | 0.0003841 | 0.024020148 |
| RP11-1055B8.7 | 17 | 376564 | | 3.3627 | 0.00038597 | 0.024057953 |
| REEP3 | 10 | 376564 | | 3.3559 | 0.0003955 | 0.024571407 |
| BRWD1 | 21 | 376564 | | 3.3546 | 0.00039741 | 0.024609647 |
| BAK1 | 6 | 376564 | | 3.3517 | 0.00040157 | 0.024786517 |
| BRPF3 | 6 | 376564 | | 3.3494 | 0.000405 | 0.02491733 |
| CSF2 | 5 | 376564 | | 3.3484 | 0.00040645 | 0.024925874 |
| SLC44A4 | 6 | 376564 | | 3.3464 | 0.00040935 | 0.025023 |
| SMAD3 | 15 | 376564 | | 3.34 | 0.00041886 | 0.025522268 |
| C1orf51 | 1 | 376564 | | 3.335 | 0.00042651 | 0.02585135 |
| CDYL2 | 16 | 376564 | | 3.3343 | 0.00042757 | 0.02585135 |
| DNAJC5 | 20 | 376564 | | 3.3332 | 0.00042929 | 0.02585135 |
| SERBP1 | 1 | 376564 | | 3.3329 | 0.0004297 | 0.02585135 |
| RBAK | 7 | 376564 | | 3.3289 | 0.0004359 | 0.026141624 |
| CEACAM21 | 19 | 376564 | | 3.3279 | 0.0004375 | 0.026155071 |
| CASC3 | 17 | 376564 | | 3.3268 | 0.00043932 | 0.026181544 |
| SARDH | 9 | 376564 | | 3.3253 | 0.00044157 | 0.026233398 |
| ADAMTS16 | 5 | 376564 | | 3.3231 | 0.00044516 | 0.026350899 |
| RCL1 | 9 | 376564 | | 3.3223 | 0.00044632 | 0.026350899 |
| NBPF20 | 1 | 376564 | | 3.3205 | 0.0004492 | 0.026438827 |
| HIST1H2AH | 6 | 376564 | | 3.3164 | 0.00045596 | 0.026753875 |
| CATSPER3 | 5 | 376564 | | 3.3152 | 0.00045795 | 0.026782255 |
| MRPL20 | 1 | 376564 | | 3.3138 | 0.00046022 | 0.026782255 |
| A3GALT2 | 1 | 376564 | | 3.3135 | 0.00046067 | 0.026782255 |
| ACTR1A | 10 | 376564 | | 3.3112 | 0.00046443 | 0.026918533 |
| FYCO1 | 3 | 376564 | | 3.3097 | 0.0004669 | 0.026941467 |
| PRKCB | 16 | 376564 | | 3.3093 | 0.00046766 | 0.026941467 |
| IDH2 | 15 | 376564 | | 3.3078 | 0.00047022 | 0.027007107 |
| BAG6 | 6 | 376564 | | 3.3057 | 0.00047375 | 0.027127895 |
| DNMT3A | 2 | 376564 | | 3.3043 | 0.00047608 | 0.02717945 |
| NARS2 | 11 | 376564 | | 3.2983 | 0.00048634 | 0.027682065 |
| CCL11 | 17 | 376564 | | 3.2927 | 0.00049624 | 0.02816125 |
| RP11-3B7.1 | 3 | 376564 | | 3.2906 | 0.00049995 | 0.028242335 |
| WDR6 | 3 | 376564 | | 3.2902 | 0.00050064 | 0.028242335 |
| HORMAD2 | 22 | 376564 | | 3.2879 | 0.00050475 | 0.028389947 |
| HLA-G | 6 | 376564 | | 3.2749 | 0.00052852 | 0.029639215 |
| MSL1 | 17 | 376564 | | 3.2724 | 0.00053322 | 0.029814839 |
| PEX13 | 2 | 376564 | | 3.2678 | 0.00054199 | 0.030160814 |
| BCL7A | 12 | 376564 | | 3.2674 | 0.00054273 | 0.030160814 |
| SLC26A6 | 3 | 376564 | | 3.2663 | 0.00054484 | 0.030160814 |
| PPT2 | 6 | 376564 | | 3.2655 | 0.00054642 | 0.030160814 |
| RNF39 | 6 | 376564 | | 3.265 | 0.00054734 | 0.030160814 |
| CHN2 | 7 | 376564 | | 3.2476 | 0.00058194 | 0.031974744 |
| NUTF2 | 16 | 376564 | | 3.245 | 0.00058715 | 0.032057775 |
| KEAP1 | 19 | 376564 | | 3.245 | 0.00058726 | 0.032057775 |
| IL12RB2 | 1 | 376564 | | 3.2444 | 0.00058851 | 0.032057775 |
| FILIP1 | 6 | 376564 | | 3.2388 | 0.00060015 | 0.032598433 |
| HSPA1B | 6 | 376564 | | 3.2326 | 0.00061331 | 0.033218337 |
| GLTPD1 | 1 | 376564 | | 3.2292 | 0.00062079 | 0.033527951 |
| DDX28 | 16 | 376564 | | 3.2271 | 0.00062518 | 0.033669397 |
| RNASET2 | 6 | 376564 | | 3.2238 | 0.00063254 | 0.033930363 |
| INSL4 | 9 | 376564 | | 3.2229 | 0.00063459 | 0.033930363 |
| RTKN2 | 10 | 376564 | | 3.2225 | 0.00063538 | 0.033930363 |
| IKZF1 | 7 | 376564 | | 3.2209 | 0.00063894 | 0.033996235 |
| KIAA1683 | 19 | 376564 | | 3.2203 | 0.00064019 | 0.033996235 |
| THRA | 17 | 376564 | | 3.2193 | 0.00064253 | 0.034025454 |
| CDCA4 | 14 | 376564 | | 3.2136 | 0.00065534 | 0.034607413 |
| ZNF347 | 19 | 376564 | | 3.2112 | 0.00066099 | 0.034809088 |
| PNPO | 17 | 376564 | | 3.2083 | 0.00066764 | 0.035062166 |
| ZBTB46 | 20 | 376564 | | 3.2002 | 0.00068663 | 0.035960118 |
| MAPK14 | 6 | 376564 | | 3.1993 | 0.0006888 | 0.035974662 |
| ANGPT4 | 20 | 376564 | | 3.1967 | 0.00069495 | 0.036196423 |
| SLC22A4 | 5 | 376564 | | 3.1939 | 0.00070191 | 0.036459046 |
| CD2BP2 | 16 | 376564 | | 3.1789 | 0.00073928 | 0.038216747 |
| GFOD2 | 16 | 376564 | | 3.1782 | 0.00074101 | 0.038216747 |
| SLC38A10 | 17 | 376564 | | 3.1779 | 0.00074178 | 0.038216747 |
| USP42 | 7 | 376564 | | 3.1736 | 0.00075277 | 0.038600507 |
| DPEP2 | 16 | 376564 | | 3.173 | 0.00075425 | 0.038600507 |
| PKNOX1 | 21 | 376564 | | 3.1726 | 0.00075532 | 0.038600507 |
| RSL1D1 | 16 | 376564 | | 3.1697 | 0.00076302 | 0.038889472 |
| PSORS1C2 | 6 | 376564 | | 3.1572 | 0.00079654 | 0.040426511 |
| TMC8 | 17 | 376564 | | 3.1568 | 0.00079743 | 0.040426511 |
| TMEM67 | 8 | 376564 | | 3.1548 | 0.00080305 | 0.040538191 |
| CTD-2267D19.3 | 17 | 376564 | | 3.1544 | 0.00080413 | 0.040538191 |
| TCAP | 17 | 376564 | | 3.1537 | 0.00080603 | 0.040538191 |
| BRF1 | 14 | 376564 | | 3.1511 | 0.00081338 | 0.040799913 |
| CLDN8 | 21 | 376564 | | 3.1499 | 0.00081669 | 0.040858141 |
| LHX6 | 9 | 376564 | | 3.1478 | 0.00082258 | 0.041044799 |
| SLC16A14 | 2 | 376564 | | 3.1427 | 0.00083694 | 0.041652006 |
| MS4A5 | 11 | 376564 | | 3.1308 | 0.00087168 | 0.043178734 |
| TRIM15 | 6 | 376564 | | 3.1306 | 0.00087216 | 0.043178734 |
| TMEM88B | 1 | 376564 | | 3.1272 | 0.00088253 | 0.043578644 |
| USP32 | 17 | 376564 | | 3.1256 | 0.00088732 | 0.043701659 |
| CD300A | 17 | 376564 | | 3.1244 | 0.00089093 | 0.043766073 |
| TNPO3 | 7 | 376564 | | 3.123 | 0.00089523 | 0.043863963 |
| CNOT11 | 2 | 376564 | | 3.1192 | 0.00090671 | 0.044300017 |
| RNF5 | 6 | 376564 | | 3.1185 | 0.00090879 | 0.044300017 |
| GPC1 | 2 | 376564 | | 3.1176 | 0.00091175 | 0.044330637 |
| PRR5 | 22 | 376564 | | 3.1154 | 0.00091844 | 0.044541997 |
| TRPM1 | 15 | 376564 | | 3.1117 | 0.00093 | 0.044987863 |
| PSORS1C1 | 6 | 376564 | | 3.107 | 0.00094507 | 0.045600827 |
| C5orf66 | 5 | 376564 | | 3.1039 | 0.000955 | 0.045963304 |
| DCTPP1 | 16 | 376564 | | 3.103 | 0.00095772 | 0.045977815 |
| CSF3 | 17 | 376564 | | 3.1001 | 0.00096738 | 0.046324587 |
| IL2 | 4 | 376564 | | 3.0964 | 0.00097948 | 0.046786167 |
| IFNA5 | 9 | 376564 | | 3.0891 | 0.0010039 | 0.047832438 |
| LL22NC03-75H12.2 | 22 | 376564 | | 3.0822 | 0.0010275 | 0.048834506 |
| LAMTOR4 | 7 | 376564 | | 3.0804 | 0.0010334 | 0.048992437 |
| PRSS16 | 6 | 376564 | | 3.0792 | 0.0010377 | 0.049073917 |
| CACNA2D2 | 3 | 376564 | | 3.0778 | 0.0010426 | 0.049092287 |
| CCL7 | 17 | 376564 | | 3.0765 | 0.0010471 | 0.049092287 |
| ARHGAP11B | 15 | 376564 | | 3.0765 | 0.0010472 | 0.049092287 |
| RANBP10 | 16 | 376564 | | 3.0758 | 0.0010497 | 0.049092287 |
| OR11H6 | 14 | 376564 | | 3.0754 | 0.001051 | 0.049092287 |
| HMGXB4 | 22 | 376564 | | 3.0737 | 0.0010571 | 0.049256196 |
| TRIM8 | 10 | 376564 | | 3.0722 | 0.0010624 | 0.049280221 |
| ZSCAN12 | 6 | 376564 | | 3.0721 | 0.0010628 | 0.049280221 |
| SEPHS2 | 16 | 376564 | | 3.0676 | 0.0010791 | 0.04983466 |
| HIST1H2AC | 6 | 376564 | | 3.0673 | 0.00108 | 0.04983466 |

**Table S2 UC risk genes identified through gene-based association studies (validation dataset (ieu-a-32))**

| **Gene** | **CHR** | | **N** | **ZSTAT** | ***P*** | ***P*_FDR_** |
| --- | --- | --- | --- | --- | --- | --- |
| C6orf47 | 6 | 27432 | | 12.263 | 7.17E-35 | 1.36E-30 |
| HCG27 | 6 | 27432 | | 8.2924 | 5.55E-17 | 5.28E-13 |
| XXbac-BPG32J3.20 | 6 | 27432 | | 7.9562 | 8.87E-16 | 5.62E-12 |
| HLA-DQA1 | 6 | 27432 | | 7.8589 | 1.94E-15 | 9.21E-12 |
| STK19 | 6 | 27432 | | 7.7608 | 4.22E-15 | 1.61E-11 |
| HLA-DRB5 | 6 | 27432 | | 7.7332 | 5.25E-15 | 1.66E-11 |
| DLD | 7 | 27432 | | 7.5771 | 1.77E-14 | 4.80E-11 |
| MST1 | 3 | 27432 | | 7.5521 | 2.14E-14 | 5.09E-11 |
| APEH | 3 | 27432 | | 7.4729 | 3.92E-14 | 8.29E-11 |
| ATF6B | 6 | 27432 | | 7.3741 | 8.27E-14 | 1.52E-10 |
| ABHD16A | 6 | 27432 | | 7.3658 | 8.80E-14 | 1.52E-10 |
| BSN | 3 | 27432 | | 7.3371 | 1.09E-13 | 1.73E-10 |
| OTUD3 | 1 | 27432 | | 7.1701 | 3.75E-13 | 5.48E-10 |
| VWA7 | 6 | 27432 | | 7.1511 | 4.30E-13 | 5.85E-10 |
| GPANK1 | 6 | 27432 | | 7.0526 | 8.78E-13 | 1.11E-09 |
| LAMB1 | 7 | 27432 | | 6.9869 | 1.41E-12 | 1.67E-09 |
| HLA-DQB1 | 6 | 27432 | | 6.9747 | 1.53E-12 | 1.71E-09 |
| PUS10 | 2 | 27432 | | 6.9548 | 1.77E-12 | 1.87E-09 |
| TCTA | 3 | 27432 | | 6.9175 | 2.30E-12 | 2.30E-09 |
| CARD9 | 9 | 27432 | | 6.8984 | 2.63E-12 | 2.46E-09 |
| EHMT2 | 6 | 27432 | | 6.8938 | 2.72E-12 | 2.46E-09 |
| NELFE | 6 | 27432 | | 6.8791 | 3.01E-12 | 2.59E-09 |
| SNAPC4 | 9 | 27432 | | 6.8733 | 3.14E-12 | 2.59E-09 |
| HLA-DRB1 | 6 | 27432 | | 6.8365 | 4.06E-12 | 3.22E-09 |
| ATP6V1G2-DDX39B | 6 | 27432 | | 6.8081 | 4.94E-12 | 3.76E-09 |
| IL23R | 1 | 27432 | | 6.7971 | 5.34E-12 | 3.90E-09 |
| C1orf106 | 1 | 27432 | | 6.7634 | 6.74E-12 | 4.64E-09 |
| NKX2-3 | 10 | 27432 | | 6.7615 | 6.83E-12 | 4.64E-09 |
| ZPBP2 | 17 | 27432 | | 6.6127 | 1.89E-11 | 1.24E-08 |
| HSPA6 | 1 | 27432 | | 6.5692 | 2.53E-11 | 1.60E-08 |
| HLA-DQB2 | 6 | 27432 | | 6.5562 | 2.76E-11 | 1.69E-08 |
| PSORS1C1 | 6 | 27432 | | 6.5393 | 3.09E-11 | 1.84E-08 |
| TCF19 | 6 | 27432 | | 6.5339 | 3.20E-11 | 1.85E-08 |
| DAG1 | 3 | 27432 | | 6.451 | 5.55E-11 | 3.11E-08 |
| NICN1 | 3 | 27432 | | 6.4443 | 5.81E-11 | 3.16E-08 |
| MICB | 6 | 27432 | | 6.3693 | 9.49E-11 | 5.02E-08 |
| C1orf141 | 1 | 27432 | | 6.2479 | 2.08E-10 | 1.07E-07 |
| HSPA1L | 6 | 27432 | | 6.2289 | 2.35E-10 | 1.18E-07 |
| SLC26A3 | 7 | 27432 | | 6.1094 | 5.00E-10 | 2.35E-07 |
| TNXB | 6 | 27432 | | 6.1094 | 5.00E-10 | 2.35E-07 |
| DDX39B | 6 | 27432 | | 6.1071 | 5.07E-10 | 2.35E-07 |
| CDSN | 6 | 27432 | | 6.0416 | 7.63E-10 | 3.46E-07 |
| RHOA | 3 | 27432 | | 5.9878 | 1.06E-09 | 4.70E-07 |
| TNFRSF6B | 20 | 27432 | | 5.9743 | 1.16E-09 | 5.00E-07 |
| RTEL1-TNFRSF6B | 20 | 27432 | | 5.9607 | 1.26E-09 | 5.31E-07 |
| SDCCAG3 | 9 | 27432 | | 5.8874 | 1.96E-09 | 8.11E-07 |
| JAK2 | 9 | 27432 | | 5.8836 | 2.01E-09 | 8.13E-07 |
| RTEL1 | 20 | 27432 | | 5.829 | 2.79E-09 | 1.10E-06 |
| TNFRSF14 | 1 | 27432 | | 5.8181 | 2.98E-09 | 1.16E-06 |
| AMT | 3 | 27432 | | 5.7924 | 3.47E-09 | 1.32E-06 |
| SLC26A6 | 3 | 27432 | | 5.7693 | 3.98E-09 | 1.48E-06 |
| NFKBIL1 | 6 | 27432 | | 5.7024 | 5.91E-09 | 2.15E-06 |
| RNF123 | 3 | 27432 | | 5.7 | 5.99E-09 | 2.15E-06 |
| ARFRP1 | 20 | 27432 | | 5.6789 | 6.78E-09 | 2.39E-06 |
| GPR35 | 2 | 27432 | | 5.6726 | 7.03E-09 | 2.43E-06 |
| FCGR2A | 1 | 27432 | | 5.611 | 1.01E-08 | 3.42E-06 |
| CELSR3 | 3 | 27432 | | 5.5864 | 1.16E-08 | 3.87E-06 |
| LY6G5C | 6 | 27432 | | 5.5662 | 1.30E-08 | 4.27E-06 |
| ZGPAT | 20 | 27432 | | 5.5363 | 1.54E-08 | 4.98E-06 |
| RP4-583P15.15 | 20 | 27432 | | 5.5167 | 1.73E-08 | 5.47E-06 |
| GPX1 | 3 | 27432 | | 5.4382 | 2.69E-08 | 8.39E-06 |
| IP6K2 | 3 | 27432 | | 5.434 | 2.76E-08 | 8.46E-06 |
| IL17REL | 22 | 27432 | | 5.3756 | 3.82E-08 | 1.15E-05 |
| NOTCH4 | 6 | 27432 | | 5.3332 | 4.82E-08 | 1.43E-05 |
| GSDMB | 17 | 27432 | | 5.3256 | 5.03E-08 | 1.47E-05 |
| LIME1 | 20 | 27432 | | 5.2821 | 6.38E-08 | 1.84E-05 |
| DNLZ | 9 | 27432 | | 5.2394 | 8.05E-08 | 2.29E-05 |
| CDHR4 | 3 | 27432 | | 5.2183 | 9.03E-08 | 2.53E-05 |
| AAMP | 2 | 27432 | | 5.173 | 1.15E-07 | 3.15E-05 |
| C3orf62 | 3 | 27432 | | 5.1715 | 1.16E-07 | 3.15E-05 |
| RP4-583P15.14 | 20 | 27432 | | 5.1547 | 1.27E-07 | 3.40E-05 |
| C9orf163 | 9 | 27432 | | 5.1424 | 1.36E-07 | 3.58E-05 |
| HLA-DOB | 6 | 27432 | | 5.1313 | 1.44E-07 | 3.75E-05 |
| NCKIPSD | 3 | 27432 | | 5.0764 | 1.92E-07 | 4.94E-05 |
| TRAIP | 3 | 27432 | | 5.0532 | 2.17E-07 | 5.45E-05 |
| CAMKV | 3 | 27432 | | 5.0529 | 2.18E-07 | 5.45E-05 |
| TAP2 | 6 | 27432 | | 5.0484 | 2.23E-07 | 5.45E-05 |
| IP6K1 | 3 | 27432 | | 5.0478 | 2.23E-07 | 5.45E-05 |
| AL161450.1 | 9 | 27432 | | 5.0329 | 2.42E-07 | 5.82E-05 |
| IL10 | 1 | 27432 | | 5.0271 | 2.49E-07 | 5.92E-05 |
| MON1A | 3 | 27432 | | 5.0178 | 2.61E-07 | 6.14E-05 |
| TMEM89 | 3 | 27432 | | 4.9871 | 3.07E-07 | 7.11E-05 |
| KLHDC8B | 3 | 27432 | | 4.9441 | 3.83E-07 | 8.77E-05 |
| PARK7 | 1 | 27432 | | 4.9262 | 4.19E-07 | 9.50E-05 |
| RBM5 | 3 | 27432 | | 4.9206 | 4.31E-07 | 9.65E-05 |
| ERRFI1 | 1 | 27432 | | 4.9149 | 4.44E-07 | 9.83E-05 |
| TMBIM1 | 2 | 27432 | | 4.9026 | 4.73E-07 | 0.000103369 |
| FAM213B | 1 | 27432 | | 4.88 | 5.30E-07 | 0.00011413 |
| SLC2A4RG | 20 | 27432 | | 4.8787 | 5.34E-07 | 0.00011413 |
| CUL2 | 10 | 27432 | | 4.8646 | 5.73E-07 | 0.000121189 |
| ORMDL3 | 17 | 27432 | | 4.8509 | 6.14E-07 | 0.000127689 |
| AMZ1 | 7 | 27432 | | 4.8499 | 6.18E-07 | 0.000127689 |
| INSL6 | 9 | 27432 | | 4.8336 | 6.71E-07 | 0.000137146 |
| PLA2G2E | 1 | 27432 | | 4.8163 | 7.31E-07 | 0.000147933 |
| ARPC2 | 2 | 27432 | | 4.8125 | 7.45E-07 | 0.000148411 |
| STMN3 | 20 | 27432 | | 4.8115 | 7.49E-07 | 0.000148411 |
| STAT3 | 17 | 27432 | | 4.7849 | 8.55E-07 | 0.000167701 |
| INSL4 | 9 | 27432 | | 4.7472 | 1.03E-06 | 0.000200109 |
| RBM6 | 3 | 27432 | | 4.7174 | 1.19E-06 | 0.000229462 |
| KIF21B | 1 | 27432 | | 4.7063 | 1.26E-06 | 0.000239137 |
| CREM | 10 | 27432 | | 4.7049 | 1.27E-06 | 0.000239137 |
| REXO2 | 11 | 27432 | | 4.6989 | 1.31E-06 | 0.00024386 |
| TAP2 | 6 | 27432 | | 4.6922 | 1.35E-06 | 0.000248663 |
| NOTCH1 | 9 | 27432 | | 4.691 | 1.36E-06 | 0.000248663 |
| CCDC36 | 3 | 27432 | | 4.6852 | 1.40E-06 | 0.00025336 |
| ATAT1 | 6 | 27432 | | 4.6742 | 1.48E-06 | 0.000263396 |
| NXPE4 | 11 | 27432 | | 4.6733 | 1.48E-06 | 0.000263396 |
| INPP5E | 9 | 27432 | | 4.6694 | 1.51E-06 | 0.000265995 |
| HLA-DQA2 | 6 | 27432 | | 4.6673 | 1.53E-06 | 0.000266242 |
| MST1R | 3 | 27432 | | 4.6543 | 1.63E-06 | 0.000280016 |
| USP4 | 3 | 27432 | | 4.6526 | 1.64E-06 | 0.000280016 |
| PNKD | 2 | 27432 | | 4.6513 | 1.65E-06 | 0.000280016 |
| CACNA1S | 1 | 27432 | | 4.6199 | 1.92E-06 | 0.000323138 |
| PPP1R10 | 6 | 27432 | | 4.6172 | 1.94E-06 | 0.000324425 |
| LST1 | 6 | 27432 | | 4.6091 | 2.02E-06 | 0.000331736 |
| CAMK2A | 5 | 27432 | | 4.609 | 2.02E-06 | 0.000331736 |
| NCR3 | 6 | 27432 | | 4.5999 | 2.11E-06 | 0.000343662 |
| BTNL2 | 6 | 27432 | | 4.5975 | 2.14E-06 | 0.000344699 |
| PMPCA | 9 | 27432 | | 4.5857 | 2.26E-06 | 0.000361607 |
| POU5F1 | 6 | 27432 | | 4.5791 | 2.34E-06 | 0.000370165 |
| CXCR2 | 2 | 27432 | | 4.5633 | 2.52E-06 | 0.000395763 |
| IRF5 | 7 | 27432 | | 4.5203 | 3.09E-06 | 0.000481309 |
| GSDMA | 17 | 27432 | | 4.5014 | 3.38E-06 | 0.000522103 |
| CFB | 6 | 27432 | | 4.4867 | 3.62E-06 | 0.000554861 |
| PSORS1C2 | 6 | 27432 | | 4.4828 | 3.68E-06 | 0.000560648 |
| MMEL1 | 1 | 27432 | | 4.481 | 3.71E-06 | 0.000560697 |
| C3orf84 | 3 | 27432 | | 4.4767 | 3.79E-06 | 0.000564493 |
| CFB | 6 | 27432 | | 4.4762 | 3.80E-06 | 0.000564493 |
| REL | 2 | 27432 | | 4.4638 | 4.03E-06 | 0.00058455 |
| ATP6V1G2 | 6 | 27432 | | 4.4634 | 4.03E-06 | 0.00058455 |
| IL2 | 4 | 27432 | | 4.4624 | 4.05E-06 | 0.00058455 |
| TNFSF15 | 9 | 27432 | | 4.4622 | 4.06E-06 | 0.00058455 |
| IMPDH2 | 3 | 27432 | | 4.4458 | 4.38E-06 | 0.000626091 |
| HLA-DMA | 6 | 27432 | | 4.4367 | 4.57E-06 | 0.000648361 |
| ZFP90 | 16 | 27432 | | 4.3857 | 5.78E-06 | 0.000814381 |
| CTD-2330K9.3 | 3 | 27432 | | 4.3598 | 6.51E-06 | 0.00091056 |
| GMEB2 | 20 | 27432 | | 4.3478 | 6.87E-06 | 0.000954438 |
| HLA-B | 6 | 27432 | | 4.3192 | 7.83E-06 | 0.001079276 |
| TMEM180 | 10 | 27432 | | 4.3041 | 8.38E-06 | 0.001147021 |
| QRICH1 | 3 | 27432 | | 4.2983 | 8.61E-06 | 0.001169343 |
| SEC16A | 9 | 27432 | | 4.2621 | 1.01E-05 | 0.001366005 |
| C6orf15 | 6 | 27432 | | 4.2545 | 1.05E-05 | 0.001403268 |
| IL21 | 4 | 27432 | | 4.2357 | 1.14E-05 | 0.001515162 |
| EGFL8 | 6 | 27432 | | 4.2221 | 1.21E-05 | 0.001598424 |
| SAPCD1 | 6 | 27432 | | 4.2173 | 1.24E-05 | 0.00162177 |
| CAMSAP2 | 1 | 27432 | | 4.1951 | 1.36E-05 | 0.00177664 |
| AC026740.1 | 5 | 27432 | | 4.1923 | 1.38E-05 | 0.001786939 |
| KIF5A | 12 | 27432 | | 4.1703 | 1.52E-05 | 0.001954536 |
| TNF | 6 | 27432 | | 4.1633 | 1.57E-05 | 0.002001801 |
| SEMA3F | 3 | 27432 | | 4.156 | 1.62E-05 | 0.00205338 |
| NOB1 | 16 | 27432 | | 4.151 | 1.66E-05 | 0.002085256 |
| MAML2 | 11 | 27432 | | 4.146 | 1.69E-05 | 0.002116837 |
| GPX6 | 6 | 27432 | | 4.1349 | 1.78E-05 | 0.002205942 |
| TNPO3 | 7 | 27432 | | 4.1336 | 1.79E-05 | 0.002205942 |
| CD274 | 9 | 27432 | | 4.1294 | 1.82E-05 | 0.002231225 |
| IL12RB2 | 1 | 27432 | | 4.1238 | 1.86E-05 | 0.0022724 |
| SFMBT1 | 3 | 27432 | | 4.1212 | 1.88E-05 | 0.002282883 |
| MIEN1 | 17 | 27432 | | 4.1157 | 1.93E-05 | 0.002323451 |
| ERBB2 | 17 | 27432 | | 4.0971 | 2.09E-05 | 0.002502876 |
| HSPA1A | 6 | 27432 | | 4.0754 | 2.30E-05 | 0.002717522 |
| TTC34 | 1 | 27432 | | 4.075 | 2.30E-05 | 0.002717522 |
| BRD7 | 16 | 27432 | | 4.0545 | 2.51E-05 | 0.002949547 |
| MAPK3 | 16 | 27432 | | 4.0148 | 2.97E-05 | 0.003471041 |
| A3GALT2 | 1 | 27432 | | 3.9961 | 3.22E-05 | 0.003733683 |
| PNMT | 17 | 27432 | | 3.9818 | 3.42E-05 | 0.003941728 |
| SCNN1D | 1 | 27432 | | 3.969 | 3.61E-05 | 0.004135005 |
| SUFU | 10 | 27432 | | 3.9522 | 3.87E-05 | 0.004410252 |
| EVX1 | 7 | 27432 | | 3.9467 | 3.96E-05 | 0.004485107 |
| EPDR1 | 7 | 27432 | | 3.9262 | 4.31E-05 | 0.004856433 |
| MED24 | 17 | 27432 | | 3.9142 | 4.53E-05 | 0.005073348 |
| GNA12 | 7 | 27432 | | 3.9054 | 4.70E-05 | 0.005230886 |
| KIAA1841 | 2 | 27432 | | 3.8991 | 4.83E-05 | 0.005339593 |
| PGAP3 | 17 | 27432 | | 3.8825 | 5.17E-05 | 0.005683101 |
| NXPE1 | 11 | 27432 | | 3.8569 | 5.74E-05 | 0.006275509 |
| LSM2 | 6 | 27432 | | 3.8467 | 5.99E-05 | 0.006505834 |
| CCHCR1 | 6 | 27432 | | 3.8422 | 6.10E-05 | 0.006588615 |
| UBA7 | 3 | 27432 | | 3.8405 | 6.14E-05 | 0.006596203 |
| RAC1 | 7 | 27432 | | 3.8317 | 6.36E-05 | 0.006799366 |
| RP11-212D19.4 | 11 | 27432 | | 3.8296 | 6.42E-05 | 0.006819825 |
| DAGLB | 7 | 27432 | | 3.8242 | 6.56E-05 | 0.006932098 |
| C2orf16 | 2 | 27432 | | 3.8208 | 6.65E-05 | 0.006990585 |
| CLSTN3 | 12 | 27432 | | 3.8186 | 6.71E-05 | 0.007012374 |
| CFDP1 | 16 | 27432 | | 3.8073 | 7.03E-05 | 0.007301777 |
| PRKD3 | 2 | 27432 | | 3.7949 | 7.39E-05 | 0.007635691 |
| GPSM1 | 9 | 27432 | | 3.774 | 8.03E-05 | 0.008258096 |
| ITGAL | 16 | 27432 | | 3.7713 | 8.12E-05 | 0.008304303 |
| ACHE | 7 | 27432 | | 3.7674 | 8.25E-05 | 0.008391007 |
| TRIP6 | 7 | 27432 | | 3.7577 | 8.57E-05 | 0.008673475 |
| PEX13 | 2 | 27432 | | 3.7442 | 9.05E-05 | 0.009104719 |
| ANKRD65 | 1 | 27432 | | 3.7336 | 9.44E-05 | 0.009434615 |
| GNAT1 | 3 | 27432 | | 3.7327 | 9.47E-05 | 0.009434615 |
| DHX16 | 6 | 27432 | | 3.7299 | 9.58E-05 | 0.009488903 |
| RP11-894J14.5 | 3 | 27432 | | 3.7285 | 9.63E-05 | 0.009491085 |
| OR5B2 | 11 | 27432 | | 3.7237 | 9.82E-05 | 0.009558926 |
| CORO1A | 16 | 27432 | | 3.7232 | 9.83E-05 | 0.009558926 |
| RP11-77K12.1 | 16 | 27432 | | 3.7228 | 9.85E-05 | 0.009558926 |
| XXbac-BPG181M17.5 | 6 | 27432 | | 3.7208 | 9.93E-05 | 0.009587163 |
| CCNL2 | 1 | 27432 | | 3.7054 | 0.00010551 | 0.010088777 |
| IKBKE | 1 | 27432 | | 3.7054 | 0.00010555 | 0.010088777 |
| CLIC1 | 6 | 27432 | | 3.7032 | 0.00010644 | 0.010122976 |
| GRID2IP | 7 | 27432 | | 3.6971 | 0.00010905 | 0.010319602 |
| LSP1 | 11 | 27432 | | 3.6919 | 0.0001113 | 0.010434377 |
| PHC2 | 1 | 27432 | | 3.6918 | 0.00011136 | 0.010434377 |
| FAIM3 | 1 | 27432 | | 3.6773 | 0.00011785 | 0.010978364 |
| C19orf12 | 19 | 27432 | | 3.6763 | 0.00011832 | 0.010978364 |
| PIM3 | 22 | 27432 | | 3.6743 | 0.00011927 | 0.01101279 |
| UBE2L3 | 22 | 27432 | | 3.6654 | 0.00012348 | 0.01134644 |
| GATA3 | 10 | 27432 | | 3.6629 | 0.00012468 | 0.011401626 |
| SLC1A5 | 19 | 27432 | | 3.6576 | 0.00012729 | 0.011584608 |
| NDUFAF7 | 2 | 27432 | | 3.654 | 0.00012908 | 0.011691575 |
| TMEM170A | 16 | 27432 | | 3.6503 | 0.00013096 | 0.011805641 |
| FGFR1OP | 6 | 27432 | | 3.6403 | 0.00013614 | 0.012214712 |
| ZBTB46 | 20 | 27432 | | 3.6291 | 0.0001422 | 0.012698527 |
| NFAT5 | 16 | 27432 | | 3.6242 | 0.00014495 | 0.012883617 |
| HLA-DOA | 6 | 27432 | | 3.6183 | 0.0001483 | 0.013120067 |
| ANKMY1 | 2 | 27432 | | 3.6143 | 0.00015061 | 0.013262744 |
| RNPEPL1 | 2 | 27432 | | 3.6124 | 0.00015168 | 0.013295416 |
| DUSP16 | 12 | 27432 | | 3.6076 | 0.00015452 | 0.013482224 |
| SERBP1 | 1 | 27432 | | 3.6058 | 0.00015559 | 0.01349367 |
| PRDM4 | 12 | 27432 | | 3.605 | 0.00015607 | 0.01349367 |
| PLCG2 | 16 | 27432 | | 3.5991 | 0.00015964 | 0.013739875 |
| RP4-758J18.2 | 1 | 27432 | | 3.5969 | 0.00016105 | 0.013798793 |
| MAPKAPK2 | 1 | 27432 | | 3.5929 | 0.00016354 | 0.013949302 |
| CXCL5 | 4 | 27432 | | 3.5901 | 0.00016528 | 0.013972019 |
| TACC1 | 8 | 27432 | | 3.5899 | 0.00016541 | 0.013972019 |
| MSH5 | 6 | 27432 | | 3.5889 | 0.00016601 | 0.013972019 |
| GPBAR1 | 2 | 27432 | | 3.5733 | 0.00017622 | 0.014747949 |
| MSH5-SAPCD1 | 6 | 27432 | | 3.5725 | 0.00017678 | 0.014747949 |
| ABHD16B | 20 | 27432 | | 3.5676 | 0.00018012 | 0.014960972 |
| LTA | 6 | 27432 | | 3.5633 | 0.00018309 | 0.015141543 |
| NRM | 6 | 27432 | | 3.5614 | 0.00018442 | 0.01518551 |
| CHP1 | 15 | 27432 | | 3.5587 | 0.00018637 | 0.01518835 |
| ITLN1 | 1 | 27432 | | 3.5583 | 0.00018664 | 0.01518835 |
| LPXN | 11 | 27432 | | 3.558 | 0.00018685 | 0.01518835 |
| ZSCAN23 | 6 | 27432 | | 3.5514 | 0.00019158 | 0.015506567 |
| INHBE | 12 | 27432 | | 3.5485 | 0.0001937 | 0.01559401 |
| ACTR1A | 10 | 27432 | | 3.5477 | 0.0001943 | 0.01559401 |
| ITPKA | 15 | 27432 | | 3.5424 | 0.00019824 | 0.015721133 |
| NUCB2 | 11 | 27432 | | 3.5421 | 0.00019847 | 0.015721133 |
| ZNF512 | 2 | 27432 | | 3.5418 | 0.0001987 | 0.015721133 |
| LPP | 3 | 27432 | | 3.5412 | 0.00019919 | 0.015721133 |
| TNP2 | 16 | 27432 | | 3.5311 | 0.0002069 | 0.016262169 |
| ABCF1 | 6 | 27432 | | 3.5234 | 0.00021301 | 0.016673511 |
| THADA | 2 | 27432 | | 3.5146 | 0.00022022 | 0.017167232 |
| VARS | 6 | 27432 | | 3.5134 | 0.00022121 | 0.017174022 |
| BRD2 | 6 | 27432 | | 3.51 | 0.0002241 | 0.017327667 |
| C20orf62 | 20 | 27432 | | 3.4976 | 0.00023469 | 0.01807303 |
| GLI1 | 12 | 27432 | | 3.4945 | 0.0002375 | 0.018215675 |
| GMPPB | 3 | 27432 | | 3.4851 | 0.00024598 | 0.018715142 |
| AMIGO3 | 3 | 27432 | | 3.4851 | 0.00024598 | 0.018715142 |
| AUH | 9 | 27432 | | 3.471 | 0.00025924 | 0.019645434 |
| C1orf222 | 1 | 27432 | | 3.4677 | 0.00026247 | 0.019811277 |
| SLC12A9 | 7 | 27432 | | 3.4664 | 0.00026373 | 0.019827701 |
| MTMR3 | 22 | 27432 | | 3.459 | 0.00027112 | 0.020303045 |
| SSTR2 | 17 | 27432 | | 3.4569 | 0.00027318 | 0.020377085 |
| PLEKHB1 | 11 | 27432 | | 3.4508 | 0.00027942 | 0.020621566 |
| IL1RL1 | 2 | 27432 | | 3.4508 | 0.00027951 | 0.020621566 |
| IL24 | 1 | 27432 | | 3.4506 | 0.00027971 | 0.020621566 |
| C6orf48 | 6 | 27432 | | 3.4466 | 0.00028389 | 0.020848925 |
| FAU | 11 | 27432 | | 3.4447 | 0.00028584 | 0.020911395 |
| EIF6 | 20 | 27432 | | 3.4431 | 0.0002875 | 0.020952251 |
| NFKB1 | 4 | 27432 | | 3.4361 | 0.0002951 | 0.021370526 |
| CEP72 | 5 | 27432 | | 3.4351 | 0.00029615 | 0.021370526 |
| RP11-3B7.1 | 3 | 27432 | | 3.4347 | 0.00029661 | 0.021370526 |
| MRPS18B | 6 | 27432 | | 3.4326 | 0.00029887 | 0.021452099 |
| AQP12A | 2 | 27432 | | 3.4137 | 0.00032049 | 0.022807085 |
| ZFP91 | 11 | 27432 | | 3.4106 | 0.00032408 | 0.022807085 |
| PUSL1 | 1 | 27432 | | 3.4103 | 0.00032441 | 0.022807085 |
| TPPP | 5 | 27432 | | 3.4102 | 0.00032458 | 0.022807085 |
| NCR3LG1 | 11 | 27432 | | 3.4092 | 0.00032574 | 0.022807085 |
| COL7A1 | 3 | 27432 | | 3.4091 | 0.00032583 | 0.022807085 |
| AC109829.1 | 2 | 27432 | | 3.4084 | 0.00032669 | 0.022807085 |
| KLF2 | 19 | 27432 | | 3.4079 | 0.00032734 | 0.022807085 |
| PTGIR | 19 | 27432 | | 3.3973 | 0.00034021 | 0.023617279 |
| FOXF1 | 16 | 27432 | | 3.386 | 0.00035465 | 0.024530173 |
| LEMD1 | 1 | 27432 | | 3.3845 | 0.0003565 | 0.024568792 |
| SLC9A4 | 2 | 27432 | | 3.3749 | 0.0003692 | 0.025352178 |
| CTXN3 | 5 | 27432 | | 3.365 | 0.00038272 | 0.026186033 |
| NQO1 | 16 | 27432 | | 3.3592 | 0.00039078 | 0.026641672 |
| BRK1 | 3 | 27432 | | 3.355 | 0.00039679 | 0.026954795 |
| NR4A1 | 12 | 27432 | | 3.3485 | 0.00040631 | 0.027503283 |
| HOXA11 | 7 | 27432 | | 3.3464 | 0.00040939 | 0.027587817 |
| IFNB1 | 9 | 27432 | | 3.3449 | 0.00041158 | 0.027587817 |
| C5orf56 | 5 | 27432 | | 3.3447 | 0.00041191 | 0.027587817 |
| QARS | 3 | 27432 | | 3.3435 | 0.00041369 | 0.027609816 |
| PSG8 | 19 | 27432 | | 3.3384 | 0.00042136 | 0.027998647 |
| CALML6 | 1 | 27432 | | 3.3376 | 0.00042246 | 0.027998647 |
| ZFP91-CNTF | 11 | 27432 | | 3.3307 | 0.0004331 | 0.02860415 |
| CLDN4 | 7 | 27432 | | 3.3244 | 0.00044299 | 0.029131544 |
| PRKD2 | 19 | 27432 | | 3.3237 | 0.00044416 | 0.029131544 |
| ABCB5 | 7 | 27432 | | 3.3227 | 0.00044568 | 0.029131544 |
| MRPL20 | 1 | 27432 | | 3.3211 | 0.00044828 | 0.029201143 |
| RTF1 | 15 | 27432 | | 3.3175 | 0.00045419 | 0.029408795 |
| PPP1R18 | 6 | 27432 | | 3.3172 | 0.00045456 | 0.029408795 |
| SLC35F6 | 2 | 27432 | | 3.3154 | 0.0004575 | 0.029498669 |
| HLA-E | 6 | 27432 | | 3.3084 | 0.0004692 | 0.030150855 |
| NME8 | 7 | 27432 | | 3.3056 | 0.00047384 | 0.030327644 |
| OR5B17 | 11 | 27432 | | 3.3048 | 0.00047514 | 0.030327644 |
| PWP1 | 12 | 27432 | | 3.3022 | 0.0004797 | 0.0305163 |
| ERAP1 | 5 | 27432 | | 3.2992 | 0.00048483 | 0.030739838 |
| FAM83C | 20 | 27432 | | 3.2973 | 0.00048816 | 0.030848144 |
| ABHD11 | 7 | 27432 | | 3.294 | 0.00049386 | 0.031105004 |
| DUSP28 | 2 | 27432 | | 3.2901 | 0.0005008 | 0.031419689 |
| FANCD2OS | 3 | 27432 | | 3.2893 | 0.00050216 | 0.031419689 |
| REV3L | 6 | 27432 | | 3.2774 | 0.00052391 | 0.032673089 |
| LRP1 | 12 | 27432 | | 3.2751 | 0.00052822 | 0.032753174 |
| C10ORF68 | 10 | 27432 | | 3.2742 | 0.00052978 | 0.032753174 |
| P4HA2 | 5 | 27432 | | 3.2739 | 0.00053036 | 0.032753174 |
| AOAH | 7 | 27432 | | 3.2726 | 0.0005328 | 0.032797375 |
| LTK | 15 | 27432 | | 3.2655 | 0.00054642 | 0.033527274 |
| CYP4V2 | 4 | 27432 | | 3.2531 | 0.00057082 | 0.034727515 |
| GLCCI1 | 7 | 27432 | | 3.2526 | 0.0005717 | 0.034727515 |
| ACADM | 1 | 27432 | | 3.252 | 0.00057302 | 0.034727515 |
| RGS14 | 5 | 27432 | | 3.2512 | 0.00057465 | 0.034727515 |
| CDKN1A | 6 | 27432 | | 3.2509 | 0.00057511 | 0.034727515 |
| YDJC | 22 | 27432 | | 3.2419 | 0.00059363 | 0.035732393 |
| C5orf66 | 5 | 27432 | | 3.2409 | 0.00059578 | 0.035748679 |
| ARAP2 | 4 | 27432 | | 3.23 | 0.00061901 | 0.037025752 |
| STAT5A | 17 | 27432 | | 3.228 | 0.00062327 | 0.037163695 |
| PTRF | 17 | 27432 | | 3.2259 | 0.00062795 | 0.03732574 |
| PPIL4 | 6 | 27432 | | 3.2229 | 0.00063459 | 0.037602917 |
| UQCRC1 | 3 | 27432 | | 3.2157 | 0.00065073 | 0.038410642 |
| TMEM52 | 1 | 27432 | | 3.215 | 0.00065226 | 0.038410642 |
| ZNF365 | 10 | 27432 | | 3.2139 | 0.00065473 | 0.038414226 |
| ZNF750 | 17 | 27432 | | 3.2132 | 0.00065636 | 0.038414226 |
| ZFP36L2 | 2 | 27432 | | 3.2119 | 0.0006592 | 0.038462096 |
| BACH2 | 6 | 27432 | | 3.2076 | 0.00066935 | 0.038934882 |
| PSMB9 | 6 | 27432 | | 3.2048 | 0.00067587 | 0.039194278 |
| TRIM8 | 10 | 27432 | | 3.2027 | 0.00068081 | 0.039360751 |
| ESPN | 1 | 27432 | | 3.1981 | 0.00069166 | 0.039866863 |
| VAMP3 | 1 | 27432 | | 3.1852 | 0.00072324 | 0.041561172 |
| LRRC3C | 17 | 27432 | | 3.1725 | 0.00075557 | 0.043288244 |
| OR5B12 | 11 | 27432 | | 3.169 | 0.00076493 | 0.043692893 |
| NADK | 1 | 27432 | | 3.1641 | 0.00077776 | 0.044292733 |
| RPRD2 | 1 | 27432 | | 3.1605 | 0.00078752 | 0.044703313 |
| CDH10 | 5 | 27432 | | 3.1597 | 0.00078967 | 0.044703313 |
| AHSA2 | 2 | 27432 | | 3.158 | 0.0007944 | 0.044837633 |
| EMC3 | 3 | 27432 | | 3.1534 | 0.00080699 | 0.045413482 |
| HES5 | 1 | 27432 | | 3.1512 | 0.00081299 | 0.045616173 |
| C6orf136 | 6 | 27432 | | 3.1495 | 0.00081784 | 0.045753337 |
| UTS2 | 1 | 27432 | | 3.1462 | 0.00082694 | 0.046126762 |
| C1orf35 | 1 | 27432 | | 3.1432 | 0.00083554 | 0.046430649 |
| STRN4 | 19 | 27432 | | 3.1426 | 0.00083727 | 0.046430649 |
| TMCO4 | 1 | 27432 | | 3.141 | 0.000842 | 0.046557215 |
| SYNGR1 | 22 | 27432 | | 3.1361 | 0.00085594 | 0.047190825 |
| GNB1 | 1 | 27432 | | 3.1297 | 0.00087479 | 0.048090695 |
| C1orf54 | 1 | 27432 | | 3.1249 | 0.00088929 | 0.048746931 |
| C1orf147 | 1 | 27432 | | 3.1225 | 0.00089654 | 0.048874705 |
| RPTN | 1 | 27432 | | 3.1225 | 0.00089676 | 0.048874705 |
| SATB2 | 2 | 27432 | | 3.1182 | 0.00090972 | 0.049439383 |

**Table S3 The significant UC risk genes identified by FUSION**

| **Gene** | **CHR** | **TWAS.Z** | **TWAS.*P*** | ***P*_FDR_** |
| --- | --- | --- | --- | --- |
| HLA-DRB1 | 6 | 10.040 | 1.01E-23 | 8.84E-20 |
| HLA-DQA2 | 6 | -9.618 | 6.68E-22 | 2.92E-18 |
| HLA-DRB6 | 6 | -9.548 | 1.32E-21 | 3.85E-18 |
| HLA-DQA1 | 6 | 9.288 | 1.57E-20 | 3.44E-17 |
| HLA-DQB2 | 6 | -8.516 | 1.64E-17 | 2.87E-14 |
| HLA-DQB1 | 6 | 8.106 | 5.21E-16 | 7.60E-13 |
| DAG1 | 3 | 8.086 | 6.12E-16 | 7.65E-13 |
| HLA-DQB1-AS1 | 6 | 8.023 | 1.03E-15 | 1.13E-12 |
| PIM3 | 22 | 6.915 | 4.65E-12 | 4.52E-09 |
| APEH | 3 | 6.682 | 2.35E-11 | 2.06E-08 |
| HLA-DRB9 | 6 | -6.378 | 1.79E-10 | 1.42E-07 |
| AC116366.6 | 5 | 6.092 | 1.11E-09 | 8.10E-07 |
| DLD | 7 | -5.952 | 2.64E-09 | 1.78E-06 |
| AF064858.8 | 21 | 5.857 | 4.69E-09 | 2.93E-06 |
| RPS23P10 | 1 | 5.765 | 8.12E-09 | 4.74E-06 |
| GPR25 | 1 | -5.676 | 1.37E-08 | 7.50E-06 |
| UBA7 | 3 | 5.652 | 1.58E-08 | 8.14E-06 |
| RP3-467L1.6 | 1 | -5.533 | 3.14E-08 | 1.53E-05 |
| ORMDL3 | 17 | -5.403 | 6.53E-08 | 3.01E-05 |
| LINC01700 | 21 | 5.364 | 8.11E-08 | 3.55E-05 |
| AF064858.11 | 21 | 5.277 | 1.31E-07 | 5.46E-05 |
| ETS2 | 21 | 5.244 | 1.57E-07 | 5.98E-05 |
| IKZF3 | 17 | 5.246 | 1.55E-07 | 5.98E-05 |
| MSH5 | 6 | -5.134 | 2.83E-07 | 0.000103 |
| PARK7 | 1 | -5.117 | 3.10E-07 | 0.000108 |
| HLA-J | 6 | 5.101 | 3.38E-07 | 0.000113 |
| TNFSF15 | 9 | -5.081 | 3.75E-07 | 0.000121 |
| CXCR1 | 2 | -4.928 | 8.28E-07 | 0.000258 |
| CXCR2 | 2 | -4.914 | 8.90E-07 | 0.000268 |
| RP11-458J1.1 | 17 | 4.828 | 1.37E-06 | 0.000399 |
| CCR3 | 3 | 4.802 | 1.57E-06 | 0.000443 |
| C6orf3 | 6 | -4.705 | 2.54E-06 | 0.000694 |
| IFITM4P | 6 | 4.620 | 3.83E-06 | 0.00101 |
| GSDMB | 17 | -4.608 | 4.05E-06 | 0.00104 |
| C5orf56 | 5 | 4.600 | 4.21E-06 | 0.00105 |
| ATG4D | 19 | -4.542 | 5.55E-06 | 0.00134 |
| RP11-378A13.1 | 2 | -4.460 | 8.17E-06 | 0.00193 |
| RTEL1 | 20 | 4.432 | 9.30E-06 | 0.00214 |
| CASC3 | 17 | -4.4151 | 1.01E-05 | 0.0022 |
| TMEM163 | 2 | 4.413 | 1.02E-05 | 0.00223 |
| IRF5 | 7 | 4.372 | 1.23E-05 | 0.00262 |
| SMARCE1 | 17 | -4.361 | 1.29E-05 | 0.00268 |
| CRIP2 | 14 | 4.306 | 1.66E-05 | 0.00337 |
| KRI1 | 19 | 4.258 | 2.06E-05 | 0.00393 |
| LIME1 | 20 | -4.257 | 2.07E-05 | 0.00393 |
| HLA-F | 6 | -4.263 | 2.01E-05 | 0.00393 |
| TAP2 | 6 | 4.237 | 2.26E-05 | 0.00420 |
| Y_RNA | 5 | -4.230 | 2.33E-05 | 0.00424 |
| CNNM2 | 10 | -4.217 | 2.47E-05 | 0.00441 |
| HIST1H4H | 6 | 4.199 | 2.67E-05 | 0.00456 |
| CCR1 | 3 | -4.196 | 2.71E-05 | 0.00456 |
| LCAT | 16 | 4.198 | 2.69E-05 | 0.00456 |
| MOG | 6 | -4.177 | 2.95E-05 | 0.00487 |
| ACD | 16 | -4.161 | 3.17E-05 | 0.00497 |
| SMG1P5 | 16 | -4.161 | 3.16E-05 | 0.00497 |
| MRPL20 | 1 | 4.160 | 3.18E-05 | 0.00497 |
| PGAP3 | 17 | -4.150 | 3.32E-05 | 0.00509 |
| SLC12A4 | 16 | 4.125 | 3.70E-05 | 0.00549 |
| VARS2 | 6 | -4.128 | 3.66E-05 | 0.00549 |
| PARD6A | 16 | -4.120 | 3.79E-05 | 0.00553 |
| AGER | 6 | -4.110 | 3.95E-05 | 0.00566 |
| TRAF3IP3 | 1 | 4.081 | 4.47E-05 | 0.00626 |
| HCG4P7 | 6 | -4.079 | 4.51E-05 | 0.00626 |
| HCG4P5 | 6 | 4.060 | 4.91E-05 | 0.00671 |
| HIST1H2BK | 6 | -4.026 | 5.67E-05 | 0.00763 |
| MED24 | 17 | 4.017 | 5.88E-05 | 0.00779 |
| ARFRP1 | 20 | -3.989 | 6.63E-05 | 0.00866 |
| NPIPB13 | 16 | -3.949 | 7.84E-05 | 0.0100 |
| CCNT2-AS1 | 2 | 3.925 | 8.66E-05 | 0.0109 |
| PNKD | 2 | -3.914 | 9.05E-05 | 0.0110 |
| PNMT | 17 | -3.917 | 8.96E-05 | 0.0110 |
| RP4-583P15.16 | 20 | 3.913 | 9.12E-05 | 0.0110 |
| OPRL1 | 20 | -3.904 | 9.45E-05 | 0.0113 |
| NELFE | 6 | -3.892 | 9.90E-05 | 0.0117 |
| RAVER1 | 19 | 3.879 | 0.000105 | 0.0120 |
| TRIM10 | 6 | 3.878 | 0.000105 | 0.0120 |
| AC002310.14 | 16 | 3.868 | 0.00011 | 0.0123 |
| RP11-122G18.11 | 1 | -3.869 | 0.000109 | 0.0123 |
| CHKB | 22 | -3.861 | 0.000113 | 0.0123 |
| EDN3 | 20 | -3.861 | 0.000113 | 0.0123 |
| RP5-832C2.5 | 1 | 3.857 | 0.000115 | 0.0124 |
| SSU72 | 1 | 3.843 | 0.000121 | 0.0129 |
| RP4-758J18.13 | 1 | -3.838 | 0.000124 | 0.0130 |
| SYNGR1 | 22 | 3.833 | 0.000126 | 0.0131 |
| STMN3 | 20 | 3.825 | 0.000131 | 0.0134 |
| ABO | 9 | 3.807 | 0.00014 | 0.014 |
| QRICH1 | 3 | -3.805 | 0.000141 | 0.0140 |
| XXbac-BPG248L24.12 | 6 | -3.807 | 0.00014 | 0.0140 |
| HCG27 | 6 | -3.779 | 0.000157 | 0.0152 |
| EIF2S2P3 | 10 | 3.781 | 0.000156 | 0.0152 |
| POLR2J3 | 7 | -3.773 | 0.000161 | 0.0154 |
| PTPRVP | 1 | -3.761 | 0.000169 | 0.0159 |
| RP11-932O9.9 | 15 | -3.761 | 0.000169 | 0.0159 |
| TMEM99 | 17 | 3.750 | 0.000177 | 0.0164 |
| NXT1 | 20 | 3.741 | 0.000183 | 0.0168 |
| CYCSP34 | 13 | -3.737 | 0.000186 | 0.0169 |
| RP11-564A8.8 | 1 | 3.730 | 0.000191 | 0.0172 |
| TMEM120A | 7 | -3.718 | 0.000201 | 0.0179 |
| DUSP28 | 2 | -3.705 | 0.000211 | 0.0184 |
| RGS17P1 | 13 | -3.706 | 0.00021 | 0.0184 |
| ARL17B | 17 | -3.700 | 0.000215 | 0.0186 |
| AP001505.10 | 21 | -3.696 | 0.000219 | 0.0187 |
| LST1 | 6 | 3.671 | 0.000241 | 0.0204 |
| CYP26B1 | 2 | -3.632 | 0.000281 | 0.0235 |
| IP6K2 | 3 | -3.628 | 0.000285 | 0.0235 |
| CCRL2 | 3 | 3.628 | 0.000285 | 0.0235 |
| FGFR1OP | 6 | 3.614 | 0.000301 | 0.0246 |
| XXbac-BPG299F13.17 | 6 | 3.607 | 0.00031 | 0.0251 |
| SLC26A6 | 3 | 3.582 | 0.00034 | 0.0273 |
| HLA-V | 6 | 3.569 | 0.000357 | 0.0284 |
| TWISTNB | 7 | -3.564 | 0.000365 | 0.0287 |
| SLC44A4 | 6 | 3.539 | 0.000401 | 0.0313 |
| RP4-591C20.9 | 20 | 3.536 | 0.000406 | 0.0314 |
| GMEB2 | 20 | -3.524 | 0.000424 | 0.0314 |
| BCL7A | 12 | -3.524 | 0.000424 | 0.0314 |
| CISD1 | 10 | 3.529 | 0.000417 | 0.0314 |
| TAS1R3 | 1 | 3.529 | 0.000417 | 0.0314 |
| HCG9 | 6 | 3.531 | 0.000414 | 0.0314 |
| KATNBL1 | 15 | -3.521 | 0.000429 | 0.0315 |
| RDX | 11 | 3.519 | 0.000433 | 0.0315 |
| RP11-309L24.10 | 7 | -3.496 | 0.000471 | 0.0340 |
| FCGR2B | 1 | 3.490 | 0.000482 | 0.0345 |
| UPK3BL | 7 | -3.470 | 0.000519 | 0.0369 |
| CALCOCO2 | 17 | -3.468 | 0.000524 | 0.0369 |
| CTRL | 16 | 3.458 | 0.000543 | 0.0380 |
| ASCC1 | 10 | -3.451 | 0.000558 | 0.038 |
| CPTP | 1 | 3.446 | 0.000568 | 0.0391 |
| MFSD13A | 10 | -3.443 | 0.000575 | 0.0393 |
| CTA-384D8.35 | 22 | -3.433 | 0.000596 | 0.0404 |
| MST1 | 3 | 3.416 | 0.000635 | 0.0426 |
| CCR9 | 3 | -3.414 | 0.000638 | 0.0426 |
| RP11-96H19.1 | 12 | -3.409 | 0.000652 | 0.0432 |
| AC034220.3 | 5 | 3.398 | 0.000677 | 0.044 |
| CDKN2D | 19 | -3.392 | 0.000692 | 0.0451 |
| WIPI2 | 7 | -3.389 | 0.000701 | 0.0451 |
| C19orf47 | 19 | 3.387 | 0.000706 | 0.0451 |
| MPI | 15 | 3.388 | 0.000702 | 0.0451 |
| SLC41A1 | 1 | 3.379 | 0.000726 | 0.0457 |
| NBPF8 | 1 | -3.381 | 0.000721 | 0.0457 |
| PPP5C | 19 | 3.372 | 0.000744 | 0.0458 |
| SGF29 | 16 | -3.373 | 0.000742 | 0.0458 |
| WASIR2 | 16 | 3.374 | 0.000738 | 0.0458 |
| MAPK3 | 16 | -3.367 | 0.000758 | 0.0461 |
| PDE8B | 5 | -3.367 | 0.000759 | 0.0461 |
| CLN3 | 16 | -3.358 | 0.000784 | 0.0471 |
| AC007193.9 | 19 | 3.357 | 0.000787 | 0.0471 |
| KIAA1683 | 19 | 3.354 | 0.000794 | 0.0472 |
| PM20D1 | 1 | -3.341 | 0.000834 | 0.0493 |

**Table S4 The intersection genes between cross-tissue and single-tissue analyses**

| **Gene** | **CHR** | **TWAS.Z** | **TWAS.*P*** | ***P*_FDR_** |
| --- | --- | --- | --- | --- |
| PIM3 | 22 | 6.9158 | 4.65E-12 | 4.52E-09 |
| CXCR2 | 2 | -4.91459 | 8.90E-07 | 0.000268 |
| TMEM163 | 2 | 4.41386 | 1.02E-05 | 0.00223 |
| CCNT2-AS1 | 2 | 3.92549 | 8.66E-05 | 0.0109 |
| PNKD | 2 | -3.91463 | 9.05E-05 | 0.0110 |
| DUSP28 | 2 | -3.70507 | 0.000211 | 0.0184 |

**Table S5 The significant genes for UC risk identified through cross-tissue analysis in the validation dataset (ieu-a-32)**

| **Gene** | **CHR** | **Test score** | ***P*** | ***P*_FDR_** |
| --- | --- | --- | --- | --- |
| COPS9 | 2 | 24.829 | 2.47E-11 | 9.22E-08 |
| PUS10 | 2 | 17.345 | 6.55E-09 | 8.16E-06 |
| FAM161A | 2 | 17.956 | 4.98E-09 | 8.16E-06 |
| CXCR2 | 2 | 15.359 | 6.63E-08 | 6.20E-05 |
| PIM3 | 22 | 14.349 | 2.16E-07 | 0.000161 |
| PNKD | 2 | 14.442 | 4.19E-07 | 0.000214 |
| WNT10A | 2 | 14.975 | 4.16E-07 | 0.000214 |
| AC021016.7 | 2 | 14.317 | 4.59E-07 | 0.000214 |
| PEX13 | 2 | 12.727 | 1.78E-06 | 0.000738 |
| CTDSP1 | 2 | 11.521 | 5.68E-06 | 0.00192 |
| ARPC2 | 2 | 11.312 | 5.35E-06 | 0.00192 |
| SATB2 | 2 | 11.861 | 8.22E-06 | 0.00256 |
| KIAA1841 | 2 | 10.835 | 9.32E-06 | 0.00267 |
| TNS1 | 2 | 10.685 | 2.19E-05 | 0.00584 |
| GCKR | 2 | 9.801 | 3.68E-05 | 0.00896 |
| BPIFC | 22 | 9.947 | 3.83E-05 | 0.00896 |
| LINC01494 | 2 | 9.873 | 5.02E-05 | 0.0110 |
| AAMP | 2 | 8.746 | 8.52E-05 | 0.0176 |
| HSPD1 | 2 | 9.477 | 0.000101 | 0.0199 |
| GPN1 | 2 | 8.475 | 0.000122 | 0.0228 |
| SLC4A1AP | 2 | 7.478 | 0.000217 | 0.0376 |
| ING5 | 2 | 7.660 | 0.000221 | 0.0376 |
| CXCR2P1 | 2 | 8.322 | 0.000239 | 0.0389 |
| LINC01460 | 2 | 7.610 | 0.000273 | 0.0425 |
| CXCR1 | 2 | 7.777 | 0.000311 | 0.0452 |
| RP11-681L4.1 | 2 | 42.302 | 0.000314 | 0.0452 |
| FOSL2 | 2 | 7.646 | 0.000344 | 0.0476 |
| RP11-973H7.1 | 18 | 6.815 | 0.000374 | 0.0499 |

**Table S6 The significant UC risk genes identified by FUSION (validation dataset (ieu-a-32))**

| **Gene** | **CHR** | **TWAS.Z** | **TWAS.*P*** | ***P*_FDR_** |
| --- | --- | --- | --- | --- |
| HLA-DRB6 | 6 | -14.93 | 2.22E-50 | 1.95E-46 |
| HLA-DQA2 | 6 | -13.46 | 2.61E-41 | 1.15E-37 |
| HLA-DRB9 | 6 | -13.05 | 6.00E-39 | 1.76E-35 |
| HLA-DRB1 | 6 | 11.73 | 8.71E-32 | 1.92E-28 |
| HLA-DQB2 | 6 | -10.28 | 8.41E-25 | 1.48E-21 |
| HLA-DQA1 | 6 | 9.99 | 1.71E-23 | 2.51E-20 |
| AGER | 6 | -9.70 | 3.14E-22 | 3.95E-19 |
| HLA-DQB1 | 6 | 9.28 | 1.67E-20 | 1.84E-17 |
| HLA-DQB1-AS1 | 6 | 7.70 | 1.38E-14 | 1.35E-11 |
| CARD9 | 9 | 7.08 | 1.49E-12 | 1.27E-09 |
| HLA-DOB | 6 | -7.07 | 1.59E-12 | 1.27E-09 |
| AF064858.8 | 21 | 6.97 | 3.22E-12 | 2.36E-09 |
| LINC01700 | 21 | 6.63 | 3.32E-11 | 2.25E-08 |
| LST1 | 6 | 6.61 | 3.82E-11 | 2.40E-08 |
| TAP2 | 6 | 6.58 | 4.65E-11 | 2.73E-08 |
| DDAH2 | 6 | 6.56 | 5.47E-11 | 3.01E-08 |
| ETS2 | 21 | 6.47 | 1.00E-10 | 5.17E-08 |
| AF064858.11 | 21 | 6.45 | 1.10E-10 | 5.38E-08 |
| RP3-395M20.8 | 1 | 6.29 | 3.10E-10 | 1.44E-07 |
| GPR25 | 1 | -6.17 | 6.62E-10 | 2.91E-07 |
| RP3-395M20.9 | 1 | -5.89 | 3.82E-09 | 1.60E-06 |
| IKZF3 | 17 | 5.86 | 4.54E-09 | 1.82E-06 |
| DLD | 7 | -5.83 | 5.65E-09 | 2.16E-06 |
| GSDMB | 17 | -5.78 | 7.49E-09 | 2.75E-06 |
| FAM213B | 1 | 5.74 | 9.45E-09 | 3.20E-06 |
| ORMDL3 | 17 | -5.75 | 9.17E-09 | 3.20E-06 |
| DAG1 | 3 | 5.53 | 3.16E-08 | 1.03E-05 |
| RPS23P10 | 1 | 5.52 | 3.37E-08 | 1.06E-05 |
| MMEL1 | 1 | 5.51 | 3.67E-08 | 1.11E-05 |
| SLC26A6 | 3 | 5.40 | 6.85E-08 | 2.01E-05 |
| RP3-467L1.6 | 1 | -5.37 | 7.77E-08 | 2.20E-05 |
| XXcos-LUCA16.1 | 3 | 5.35 | 8.77E-08 | 2.41E-05 |
| LIME1 | 20 | -5.31 | 1.08E-07 | 2.88E-05 |
| PIM3 | 22 | 5.25 | 1.52E-07 | 3.93E-05 |
| IRF5 | 7 | 5.21 | 1.94E-07 | 4.68E-05 |
| TMEM89 | 3 | 5.20 | 1.97E-07 | 4.68E-05 |
| STMN3 | 20 | 5.21 | 1.93E-07 | 4.68E-05 |
| RP4-583P15.16 | 20 | 5.14 | 2.81E-07 | 6.50E-05 |
| MST1R | 3 | 5.10 | 3.34E-07 | 7.53E-05 |
| IFITM4P | 6 | 5.08 | 3.71E-07 | 8.16E-05 |
| RP4-591C20.9 | 20 | 5.03 | 4.92E-07 | 0.000105552 |
| ARFRP1 | 20 | -5.02 | 5.21E-07 | 0.000109112 |
| RP11-378A13.1 | 2 | -4.94 | 7.79E-07 | 0.000159351 |
| CTD-2589H19.6 | 5 | 4.93 | 8.40E-07 | 0.000167924 |
| UBA7 | 3 | 4.88 | 1.08E-06 | 0.000211104 |
| HLA-DRB5 | 6 | 4.86 | 1.15E-06 | 0.0002199 |
| SEC16A | 9 | -4.85 | 1.24E-06 | 0.000232065 |
| RTEL1 | 20 | 4.83 | 1.38E-06 | 0.000252885 |
| CXCR1 | 2 | -4.80 | 1.55E-06 | 0.000278241 |
| TNFSF15 | 9 | -4.79 | 1.63E-06 | 0.00028675 |
| MARS | 12 | -4.78 | 1.74E-06 | 0.000300099 |
| GNA12 | 7 | -4.77 | 1.83E-06 | 0.00030786 |
| RAVER1 | 19 | 4.77 | 1.87E-06 | 0.00030786 |
| C4A | 6 | 4.76 | 1.89E-06 | 0.00030786 |
| HSPA1B | 6 | 4.74 | 2.15E-06 | 0.000343844 |
| CXCR2 | 2 | -4.71 | 2.45E-06 | 0.000384825 |
| MFSD13A | 10 | -4.67 | 3.01E-06 | 0.000464491 |
| PARK7 | 1 | -4.66 | 3.23E-06 | 0.000489846 |
| HLA-DOA | 6 | 4.59 | 4.48E-06 | 0.0006679 |
| ZFP90 | 16 | 4.58 | 4.75E-06 | 0.00069635 |
| TTC34 | 1 | -4.55 | 5.35E-06 | 0.000771452 |
| SMIM25 | 20 | 4.51 | 6.34E-06 | 0.000899462 |
| SDCCAG3 | 9 | -4.46 | 8.16E-06 | 0.001133859 |
| ATP6V1G2 | 6 | -4.46 | 8.25E-06 | 0.001133859 |
| BRD7 | 16 | -4.45 | 8.44E-06 | 0.001142127 |
| CTD-3184A7.4 | 20 | 4.42 | 1.00E-05 | 0.001332727 |
| MICB | 6 | 4.36 | 1.28E-05 | 0.00168043 |
| PBX2 | 6 | 4.36 | 1.32E-05 | 0.001707459 |
| PNMT | 17 | -4.33 | 1.46E-05 | 0.001861183 |
| PRKCB | 16 | 4.33 | 1.52E-05 | 0.001909989 |
| RP11-309L24.10 | 7 | -4.30 | 1.73E-05 | 0.002143251 |
| PGAP3 | 17 | -4.28 | 1.84E-05 | 0.002247867 |
| SUFU | 10 | -4.26 | 2.07E-05 | 0.002494208 |
| RWDD1 | 6 | 4.23 | 2.34E-05 | 0.002744352 |
| CFDP1 | 16 | 4.23 | 2.34E-05 | 0.002744352 |
| PNKD | 2 | -4.14 | 3.40E-05 | 0.003935053 |
| RBM6 | 3 | 4.13 | 3.68E-05 | 0.004007067 |
| CASC3 | 17 | -4.13 | 3.58E-05 | 0.004007067 |
| ACADM | 1 | -4.13 | 3.69E-05 | 0.004007067 |
| APEH | 3 | 4.13 | 3.58E-05 | 0.004007067 |
| NFIL3 | 9 | -4.13 | 3.69E-05 | 0.004007067 |
| RNF5 | 6 | 4.11 | 3.99E-05 | 0.004280005 |
| NONOP2 | 2 | -4.09 | 4.31E-05 | 0.004567561 |
| MUSTN1 | 3 | -4.07 | 4.71E-05 | 0.004932043 |
| AC010883.5 | 2 | 4.04 | 5.31E-05 | 0.005494913 |
| DAGLB | 7 | -4.02 | 5.79E-05 | 0.005921958 |
| RP5-1057I20.6 | 12 | -3.99 | 6.59E-05 | 0.006662717 |
| WNT11 | 11 | -3.98 | 6.84E-05 | 0.006836891 |
| AAGAB | 15 | -3.97 | 7.18E-05 | 0.007096099 |
| MRPL20 | 1 | 3.96 | 7.41E-05 | 0.00724204 |
| ZMAT5 | 22 | 3.92 | 8.71E-05 | 0.008419029 |
| HCG4P7 | 6 | -3.91 | 9.07E-05 | 0.008671709 |
| NFE2L3 | 7 | -3.90 | 9.68E-05 | 0.009155406 |
| RPRD2 | 1 | -3.89 | 0.000101 | 0.009451021 |
| ITLN1 | 1 | 3.88 | 0.000105 | 0.009620625 |
| RP11-672A2.4 | 11 | -3.88 | 0.000104 | 0.009620625 |
| ITPKA | 15 | -3.87 | 0.000108 | 0.009793485 |
| MMP24-AS1 | 20 | -3.87 | 0.00011 | 0.009873061 |
| ADCY7 | 16 | -3.86 | 0.000112 | 0.00995103 |
| GMEB2 | 20 | -3.85 | 0.000116 | 0.010102337 |
| INPP5E | 9 | -3.86 | 0.000115 | 0.010102337 |
| UBE2L3 | 22 | 3.84 | 0.000123 | 0.010606941 |
| SLC22A5 | 5 | -3.81 | 0.000138 | 0.011784932 |
| BATF3 | 1 | -3.79 | 0.000148 | 0.012517385 |
| SLC22A4 | 5 | -3.78 | 0.000156 | 0.013068343 |
| CARHSP1 | 16 | 3.76 | 0.000168 | 0.013892748 |
| RP11-564A8.8 | 1 | 3.76 | 0.000169 | 0.013892748 |
| ITIH4 | 3 | -3.75 | 0.00018 | 0.01466 |
| GALC | 14 | -3.74 | 0.000184 | 0.014848294 |
| DXO | 6 | 3.73 | 0.000193 | 0.015432982 |
| MICD | 6 | 3.72 | 2.00E-04 | 0.015848649 |
| NCR3 | 6 | 3.70 | 0.000216 | 0.016963714 |
| DDX39B | 6 | -3.69 | 0.000225 | 0.017514159 |
| MST1 | 3 | 3.67 | 0.00024 | 0.018517895 |
| RP11-1149M10.2 | 8 | -3.66 | 0.000256 | 0.019580661 |
| LMAN2 | 5 | 3.64 | 0.000277 | 0.021004241 |
| GLDC | 9 | -3.62 | 0.000292 | 0.021766373 |
| UQCR10 | 22 | 3.62 | 0.000291 | 0.021766373 |
| SMG1P5 | 16 | -3.61 | 0.000306 | 0.022618286 |
| RP11-493E12.2 | 2 | 3.60 | 0.000319 | 0.0233827 |
| AFF3 | 2 | 3.58 | 0.000339 | 0.024643339 |
| TCTEX1D1 | 1 | -3.58 | 0.000346 | 0.024946033 |
| NFKB1 | 4 | 3.57 | 0.000353 | 0.025243805 |
| RTF1 | 15 | -3.57 | 0.000356 | 0.025253032 |
| HLA-F | 6 | -3.57 | 0.000363 | 0.025543584 |
| NNMT | 11 | -3.56 | 0.000366 | 0.025550286 |
| OIP5-AS1 | 15 | 3.55 | 0.000383 | 0.02652652 |
| KDELR2 | 7 | 3.53 | 0.000413 | 0.028380844 |
| TXN | 9 | -3.51 | 0.000448 | 0.029628632 |
| HLA-S | 6 | -3.51 | 0.000444 | 0.029628632 |
| RPL23AP1 | 6 | -3.52 | 0.000436 | 0.029628632 |
| BRK1 | 3 | 3.51 | 0.000446 | 0.029628632 |
| CTB-12A17.2 | 19 | -3.51 | 0.000445 | 0.029628632 |
| GDPD3 | 16 | -3.51 | 0.000455 | 0.029710933 |
| RP4-614O4.13 | 20 | -3.51 | 0.000456 | 0.029710933 |
| NPIPB7 | 16 | -3.50 | 0.000468 | 0.030268588 |
| PRDM4 | 12 | -3.49 | 0.000486 | 0.031040957 |
| CXCL5 | 4 | -3.49 | 0.000487 | 0.031040957 |
| NUCB2 | 11 | 3.48 | 0.000496 | 0.031095718 |
| NFATC1 | 18 | -3.48 | 0.000495 | 0.031095718 |
| AK3 | 9 | 3.48 | 0.000502 | 0.031095718 |
| FIS1 | 7 | -3.48 | 0.000502 | 0.031095718 |
| OS9 | 12 | 3.47 | 0.000512 | 0.031493371 |
| AC007278.2 | 2 | -3.47 | 0.000526 | 0.032129833 |
| TSPO | 22 | 3.46 | 0.000547 | 0.03274727 |
| ACADS | 12 | -3.46 | 0.000548 | 0.03274727 |
| MSH5 | 6 | -3.45 | 0.000551 | 0.03274727 |
| XXbac-BPG252P9.10 | 6 | -3.46 | 0.00055 | 0.03274727 |
| FAM183DP | 2 | 3.43 | 6.00E-04 | 0.035420134 |
| KIR2DL1 | 19 | 3.42 | 0.000628 | 0.03682592 |
| SLC25A11 | 17 | 3.41 | 0.000649 | 0.037556605 |
| FANCD2 | 3 | 3.41 | 0.000647 | 0.037556605 |
| NME8 | 7 | -3.40 | 0.000676 | 0.038321427 |
| ABHD11 | 7 | -3.40 | 0.00068 | 0.038321427 |
| IL24 | 1 | 3.40 | 0.000683 | 0.038321427 |
| NPIPB6 | 16 | 3.40 | 0.000684 | 0.038321427 |
| RP11-534L20.5 | 1 | 3.40 | 0.000684 | 0.038321427 |
| IRGM | 5 | -3.39 | 0.000694 | 0.038635595 |
| RP11-1020A11.2 | 3 | 3.39 | 0.000706 | 0.039056453 |
| TLR3 | 4 | -3.38 | 0.000722 | 0.03969195 |
| IRF6 | 1 | -3.38 | 0.000732 | 0.039991752 |
| CYP19A1 | 15 | -3.36 | 0.000785 | 0.042622593 |
| KIR3DL1 | 19 | 3.35 | 0.000809 | 0.043340291 |
| CDCA4 | 14 | 3.35 | 0.000808 | 0.043340291 |
| ABHD11-AS1 | 7 | -3.35 | 0.000813 | 0.043340291 |
| INCA1 | 17 | -3.34 | 0.00083 | 0.043716647 |
| HLA-J | 6 | 3.34 | 0.000826 | 0.043716647 |
| NFAT5 | 16 | 3.33 | 0.00087 | 0.045014824 |
| STAC3 | 12 | 3.33 | 0.000866 | 0.045014824 |
| AC116366.6 | 5 | 3.33 | 0.000864 | 0.045014824 |
| FGFR1OP | 6 | 3.33 | 0.000876 | 0.045060211 |
| CLN3 | 16 | -3.32 | 0.000912 | 0.046639256 |
| TAB2 | 6 | -3.31 | 0.000937 | 0.047640763 |
| GATS | 7 | -3.31 | 0.000948 | 0.047923034 |
| ARPC2 | 2 | -3.30 | 0.000961 | 0.048302606 |
| FAM26F | 6 | 3.30 | 0.00098 | 0.048977727 |
| SLC35F3 | 1 | -3.29 | 0.00101 | 0.049909888 |
| RP4-758J18.13 | 1 | -3.29 | 0.00101 | 0.049909888 |

**Table S7 The intersection genes between cross-tissue and single-tissue analyses (validation dataset (ieu-a-32))**

| **Gene** | **CHR** | **TWAS.Z** | **TWAS.*P*** | ***P*_FDR_** |
| --- | --- | --- | --- | --- |
| PIM3 | 22 | 5.25041 | 1.52E-07 | 3.93E-05 |
| CXCR1 | 2 | -4.80405 | 1.55E-06 | 0.000278 |
| CXCR2 | 2 | -4.71216 | 2.45E-06 | 0.000384 |
| PNKD | 2 | -4.14467 | 3.40E-05 | 0.00393 |
| ARPC2 | 2 | -3.30182 | 0.000961 | 0.0483 |

| **Gene** | **TWAS.Z** | **TWAS.*P*** | **JOINT.Z** | **JOINT.*P*** |
| --- | --- | --- | --- | --- |
| CXCR1 | -4.8 | 1.50E-06 | -4.8 | 1.60E-06 |
| PIM3 | 5.3 | 1.50E-07 | 5.3 | 1.50E-07 |

**Table S8 The significant genes for UC risk in Conditional and joint analysis (validation dataset (ieu-a-32))**

| **Gene** | **CHR** | **Ldregion pop1** | **Twas z pop1** | **Pips pop1** | **In cred set pop1** |
| --- | --- | --- | --- | --- | --- |
| DLD | 7 | 7:105683380-7:107780130 | -5.92 | 1 | 1 |
| ENSG00000269925.1 | 1 | 1:7247335-1:9365093 | -5.66 | 0.965 | 1 |
| GPR25 | 1 | 1:200137649-1:201589075 | -5.51 | 1 | 1 |
| C10orf95-AS1 | 10 | 10:102949821-10:104380169 | -5.35 | 0.998 | 1 |
| HAR1B | 20 | 20:62190180-20:62963102 | -5.1 | 0.982 | 1 |
| TNFSF15 | 9 | 9:117019801-9:117921871 | -4.84 | 0.999 | 1 |
| OCM | 7 | 7:5416232-7:5854526 | -4.42 | 0.973 | 1 |
| MICB | 6 | 6:31571337-6:32682664 | -1.1 | 0.926 | 1 |
| TAP2 | 6 | 6:32682664-6:33236497 | 4.14 | 0.89 | 1 |
| ENSG00000274501.1 | 20 | 20:62190180-20:62963102 | 4.21 | 0.863 | 1 |
| IRF5 | 7 | 7:126869221-7:128778386 | 4.27 | 0.914 | 1 |
| SPATA6L | 9 | 9:4885082-9:6557589 | 4.86 | 0.972 | 1 |
| RPS23P10 | 1 | 1:159913048-1:162346607 | 5.36 | 0.998 | 1 |
| ENSG00000234290.2 | 5 | 5:129519025-5:132139647 | 6.01 | 0.997 | 1 |
| PIM3 | 22 | 22:49825112-22:51240820 | 6.85 | 1 | 1 |
| DAG1 | 3 | 3:49317338-3:51830565 | 8.09 | 1 | 1 |
| HLA-DRB1 | 6 | 6:31571337-6:32682664 | 11.3 | 1 | 1 |

**Table S9 The results of FOCUS precision positioning**

**Table S10 The results of FOCUS precision positioning (validation dataset (ieu-a-32))**

| **Gene** | **CHR** | **Ldregion pop1** | **Twas z pop1** | **Pips pop1** | **In cred set pop1** |
| --- | --- | --- | --- | --- | --- |
| QRICH1 | 3 | 3:49317338-3:51830565 | -6.79 | 0.937 | 1 |
| GPR25 | 1 | 1:200137649-1:201589075 | -6 | 1 | 1 |
| DLD | 7 | 7:105683380-7:107780130 | -5.79 | 1 | 1 |
| ENSG00000269925.1 | 1 | 1:7247335-1:9365093 | -5.6 | 0.994 | 1 |
| HLA-DRB6 | 6 | 6:32682664-6:33236497 | -4.98 | 0.953 | 1 |
| GNA12 | 7 | 7:2772261-7:4573169 | -4.83 | 0.999 | 1 |
| TNFSF15 | 9 | 9:117019801-9:117921871 | -4.57 | 0.998 | 1 |
| NONOP2 | 2 | 2:60292000-2:62428117 | -4.32 | 0.935 | 1 |
| FAM72A | 1 | 1:206074070-1:208410160 | -3.33 | 0.849 | 1 |
| IKBKE-AS1 | 1 | 1:206074070-1:208410160 | 1.45 | 0.806 | 1 |
| IFITM4P | 6 | 6:28917832-6:29737971 | 4.72 | 0.899 | 1 |
| RPS23P10 | 1 | 1:159913048-1:162346607 | 5.13 | 0.997 | 1 |
| PIM3 | 22 | 22:49825112-22:51240820 | 5.19 | 0.999 | 1 |
| SLC26A6 | 3 | 3:47727379-3:49316164 | 5.35 | 0.89 | 1 |
| TNFRSF14-AS1 | 1 | 1:1893006-1:3582374 | 6.16 | 0.936 | 1 |
| TAP2 | 6 | 6:32682664-6:33236497 | 6.42 | 0.815 | 1 |
| LINC02940 | 21 | 21:38711704-21:40480359 | 6.9 | 0.92 | 1 |
| CARD9 | 9 | 9:137589364-9:141124247 | 6.95 | 1 | 1 |
| LST1 | 6 | 6:31571337-6:32682664 | 9.86 | 1 | 1 |

**Table S11 The intersection gene from four genetic analyses**

| **Gene** | **Gene id** | **CHR** | **PPH4** |
| --- | --- | --- | --- |
| PIM3 | ENSG00000198355 | 22 | 0.997 |

**Table S12 SNPs for MR filtered by criteria (*P* < 5E-08)**

| **SNP** | **Chr** | **Pos** | **Effect allele** | **Other allele** | **Eaf** | **Beta** | **Se** | ***P*** |
| --- | --- | --- | --- | --- | --- | --- | --- | --- |
| rs28634677 | 22 | 50351338 | G | C | 0.225373 | -0.196377 | 0.0303086 | 1.92E-10 |
| rs28681372 | 22 | 50351977 | A | G | 0.387313 | -0.197458 | 0.0257464 | 7.01E-14 |
| rs28496728 | 22 | 50352623 | G | A | 0.270149 | 0.152438 | 0.0270742 | 2.76E-08 |
| rs56150947 | 22 | 50353919 | A | G | 0.445522 | -0.261909 | 0.0248013 | 5.02E-24 |
| rs28424067 | 22 | 50354011 | A | G | 0.248507 | 0.156444 | 0.0279902 | 3.46E-08 |
| rs28375915 | 22 | 50354255 | A | G | 0.475373 | -0.246877 | 0.0239346 | 4.48E-23 |
| rs111752560 | 22 | 50354272 | C | A | 0.5 | -0.24828 | 0.024294 | 1.03E-22 |
| rs28645887 | 22 | 50356274 | T | C | 0.485821 | -0.25892 | 0.0239853 | 6.00E-25 |
| rs28691713 | 22 | 50356302 | T | C | 0.496269 | -0.255009 | 0.0240581 | 3.52E-24 |
| rs35958120 | 22 | 50361397 | G | C | 0.491045 | -0.246021 | 0.0243014 | 2.40E-22 |
| rs35407109 | 22 | 50363794 | T | C | 0.323881 | 0.190766 | 0.0256978 | 3.93E-13 |
| rs73181194 | 22 | 50363889 | A | G | 0.226866 | -0.207332 | 0.02973 | 8.17E-12 |
| rs36147371 | 22 | 50414983 | T | C | 0.479104 | -0.245484 | 0.0242934 | 2.82E-22 |
| rs36195165 | 22 | 50415900 | C | T | 0.322388 | 0.192142 | 0.0254261 | 1.55E-13 |
| rs36183197 | 22 | 50416205 | A | C | 0.314179 | 0.187463 | 0.0256652 | 8.92E-13 |
| rs36150983 | 22 | 50416247 | A | G | 0.314179 | 0.187463 | 0.0256652 | 8.92E-13 |
| rs140184333 | 22 | 50416718 | T | C | 0.321642 | 0.19289 | 0.0253895 | 1.17E-13 |
| rs36163375 | 22 | 50417258 | G | A | 0.314179 | -0.187463 | 0.0256652 | 8.92E-13 |
| rs36155743 | 22 | 50417483 | T | C | 0.478358 | -0.245388 | 0.0242811 | 2.80E-22 |
| rs144554206 | 22 | 50418996 | A | G | 0.45597 | -0.253487 | 0.0249647 | 1.84E-22 |
| rs145095295 | 22 | 50419266 | T | C | 0.493284 | -0.239159 | 0.0243852 | 3.68E-21 |
| rs36138276 | 22 | 50422348 | A | G | 0.475373 | -0.247698 | 0.0246174 | 4.11E-22 |
| rs36171610 | 22 | 50422385 | G | A | 0.492537 | -0.244364 | 0.0242753 | 3.95E-22 |
| rs141216971 | 22 | 50422420 | G | C | 0.493994 | -0.245108 | 0.0243804 | 4.42E-22 |
| rs36168477 | 22 | 50424400 | T | C | 0.497761 | -0.249132 | 0.0246121 | 2.43E-22 |
| rs148032669 | 22 | 50424893 | C | T | 0.491045 | -0.252391 | 0.024522 | 5.45E-23 |
| rs4838881 | 22 | 50425779 | T | C | 0.497015 | -0.249041 | 0.0245661 | 2.12E-22 |
| rs137834 | 22 | 50426987 | A | G | 0.497761 | 0.249554 | 0.024511 | 1.45E-22 |
| rs137835 | 22 | 50428069 | G | A | 0.496269 | 0.242194 | 0.0244067 | 1.36E-21 |
| rs137837 | 22 | 50428382 | T | C | 0.497015 | 0.249041 | 0.0245661 | 2.12E-22 |
| rs137838 | 22 | 50428839 | C | T | 0.486567 | 0.247801 | 0.0244646 | 2.29E-22 |
| rs137839 | 22 | 50429332 | A | C | 0.264179 | 0.161709 | 0.028085 | 1.36E-08 |
| rs137840 | 22 | 50431414 | T | C | 0.499254 | 0.244922 | 0.0246303 | 1.14E-21 |
| rs137841 | 22 | 50432604 | A | C | 0.276119 | 0.155177 | 0.0276552 | 3.07E-08 |
| rs137843 | 22 | 50434619 | C | G | 0.376119 | 0.16584 | 0.0258333 | 2.78E-10 |
| rs5771069 | 22 | 50435480 | G | A | 0.479104 | 0.242583 | 0.0243998 | 1.16E-21 |
| rs4838836 | 22 | 50437202 | G | C | 0.323881 | 0.176106 | 0.0254426 | 1.15E-11 |
| rs4838838 | 22 | 50437372 | A | G | 0.323134 | 0.176518 | 0.0254137 | 9.82E-12 |
| rs144306366 | 22 | 50437527 | GCA | G | 0.31583 | 0.176005 | 0.0261418 | 3.90E-11 |
| rs7290572 | 22 | 50437976 | T | G | 0.323881 | 0.173777 | 0.0254469 | 2.10E-11 |
| rs9617090 | 22 | 50439194 | T | C | 0.391629 | -0.235036 | 0.0258281 | 1.32E-18 |
| rs137845 | 22 | 50439430 | G | A | 0.48209 | 0.244017 | 0.0245574 | 1.22E-21 |
| rs137846 | 22 | 50439755 | GCA | G | 0.48209 | 0.244017 | 0.0245574 | 1.22E-21 |
| rs137848 | 22 | 50440296 | C | T | 0.476866 | 0.245635 | 0.0246336 | 8.99E-22 |
| rs13056642 | 22 | 50441521 | T | C | 0.289552 | 0.164895 | 0.0263501 | 7.43E-10 |
| rs5771192 | 22 | 50441985 | C | A | 0.485075 | 0.243568 | 0.0246906 | 2.25E-21 |
| rs137853 | 22 | 50443449 | T | C | 0.484328 | 0.239678 | 0.0247866 | 1.19E-20 |
| rs137854 | 22 | 50444043 | G | C | 0.485075 | 0.237636 | 0.0248724 | 3.14E-20 |
| rs137855 | 22 | 50444066 | A | G | 0.485075 | 0.237636 | 0.0248724 | 3.14E-20 |
| rs137856 | 22 | 50444484 | G | A | 0.471642 | 0.236427 | 0.0246116 | 2.02E-20 |
| rs137857 | 22 | 50444577 | G | A | 0.466418 | 0.237091 | 0.024834 | 3.33E-20 |
| rs137858 | 22 | 50445601 | C | T | 0.483582 | 0.236563 | 0.0250181 | 7.14E-20 |
| rs5771222 | 22 | 50445704 | C | T | 0.483582 | 0.236563 | 0.0250181 | 7.14E-20 |
| rs137859 | 22 | 50445922 | T | C | 0.482836 | 0.238629 | 0.0250447 | 3.90E-20 |
| rs66520758 | 22 | 50445966 | AT | A | 0.347761 | 0.154568 | 0.0279182 | 4.62E-08 |
| rs111231752 | 22 | 50446282 | TAAAC | T | 0.483582 | 0.236563 | 0.0250181 | 7.14E-20 |
| rs137862 | 22 | 50446550 | A | C | 0.484328 | 0.237319 | 0.0249684 | 4.74E-20 |
| rs137864 | 22 | 50446988 | T | C | 0.488806 | 0.239845 | 0.0249059 | 1.66E-20 |
| rs137865 | 22 | 50447354 | C | T | 0.318657 | 0.157453 | 0.0257786 | 1.82E-09 |
| rs137866 | 22 | 50447384 | A | G | 0.298507 | 0.166799 | 0.0258161 | 2.15E-10 |
| rs71196396 | 22 | 50449433 | T | TC | 0.481331 | 0.158915 | 0.0277254 | 1.57E-08 |
| rs73183310 | 22 | 50449531 | G | A | 0.30597 | -0.179058 | 0.0273519 | 1.27E-10 |
| rs5771254 | 22 | 50449762 | C | A | 0.357463 | -0.156473 | 0.0269153 | 9.94E-09 |
| rs12171069 | 22 | 50450133 | C | G | 0.213433 | -0.183048 | 0.0302131 | 2.43E-09 |
| rs137869 | 22 | 50450491 | T | C | 0.484328 | 0.179055 | 0.0252176 | 3.53E-12 |
| rs66498467 | 22 | 50450508 | C | CTGAG | 0.484328 | 0.179055 | 0.0252176 | 3.53E-12 |
| rs137871 | 22 | 50450543 | T | C | 0.484328 | 0.179055 | 0.0252176 | 3.53E-12 |
| rs137872 | 22 | 50451279 | C | CG | 0.455224 | 0.194911 | 0.0251409 | 3.87E-14 |
| rs137873 | 22 | 50451687 | C | T | 0.484328 | 0.17943 | 0.0251071 | 2.59E-12 |
| rs713669 | 22 | 50451771 | T | C | 0.459701 | 0.198013 | 0.0250573 | 1.31E-14 |
| rs137875 | 22 | 50451951 | G | A | 0.492537 | 0.177777 | 0.0250461 | 3.59E-12 |
| rs137876 | 22 | 50452207 | G | A | 0.487313 | 0.184747 | 0.0251215 | 6.34E-13 |
| rs5771120 | 22 | 50454499 | G | C | 0.31194 | -0.1635 | 0.0274931 | 4.64E-09 |
| rs137879 | 22 | 50457041 | T | C | 0.490299 | 0.15886 | 0.0255543 | 9.52E-10 |
| rs62231924 | 22 | 50468122 | T | G | 0.164179 | -0.19227 | 0.0339106 | 2.22E-08 |
